# Ez-Metastasizing: The Crucial Roles of Ezrin in Metastasis

**DOI:** 10.3390/cells12121620

**Published:** 2023-06-14

**Authors:** Rand Gabriel M. Buenaventura, Glenn Merlino, Yanlin Yu

**Affiliations:** Laboratory of Cancer Biology and Genetics, Center for Cancer Research, National Cancer Institute, National Institutes of Health, Bethesda, MD 20892, USA

**Keywords:** Ezrin, binding protein, cell migration, tumor metastasis, therapeutic target

## Abstract

Ezrin is the cytoskeletal organizer and functions in the modulation of membrane–cytoskeleton interaction, maintenance of cell shape and structure, and regulation of cell–cell adhesion and movement, as well as cell survival. Ezrin plays a critical role in regulating tumor metastasis through interaction with other binding proteins. Notably, Ezrin has been reported to interact with immune cells, allowing tumor cells to escape immune attack in metastasis. Here, we review the main functions of Ezrin, the mechanisms through which it acts, its role in tumor metastasis, and its potential as a therapeutic target.

## 1. Introduction

In 1983, Ezrin was discovered and initially characterized as a small element of the microvilli at chicken intestinal epithelial cell brush borders [1]. In later studies investigating similar proteins in actin-based cytoskeleton structures, proteins such as cytovillin [2,3], p81 [4], and 80K [5,6] were all identified and subsequently established as the same protein [7,8,9,10,11]. In the coming years, Ezrin was shown to be a key player in linking the plasma membrane to the cytoskeleton [12]. It has been well documented that Ezrin participates in various cellular processes such as signal transduction [13], cell proliferation [14], cell–cell adhesion [15,16], membrane projections [17,18,19], and cell motility [20,21], among others.

**Figure 1:**
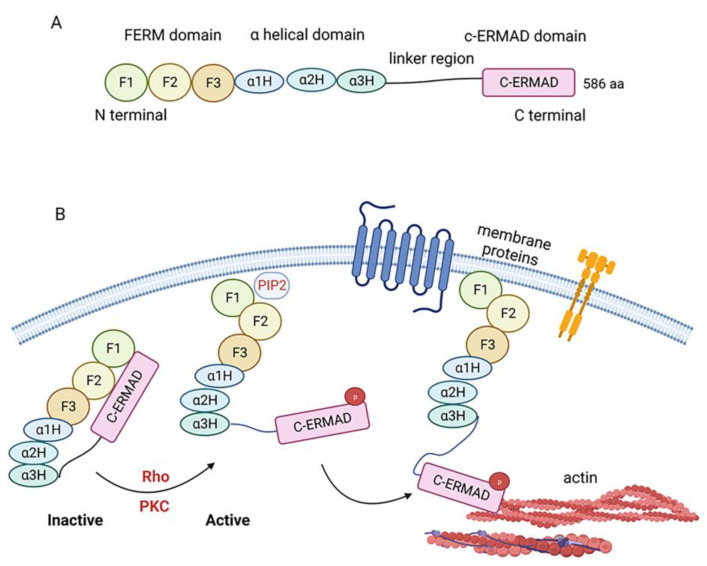
Ezrin is a linker between the cytoskeleton and cell membranes. (**A**) Schematic of the Ezrin protein. Ezrin is a protein with 586 amino acids and consists of FERM, α-helical and c-ERMAD domains, and a linker region. (**B**) Ezrin interconverts from an inactive closed conformation to an active open conformation dynamically. When Ezrin is recruited to the plasma membrane and binds to PIP_2_ at its FERM domain, c-ERMAD is released, allowing kinases such as Rho or PKC to phosphorylate the c terminal at Thr567, thereby converting Ezrin to the active form capable of binding to actin.

Ezrin, part of the ezrin/radixin/moesin (ERM) family of proteins, is encoded by the *EZR* gene. Ezrin contains three major domains: the amino-terminal FERM (four-point one, Ezrin, Radixin, Moesin) domain, the α-helical domain, and the carboxy-terminal ERM association domain (C-ERMAD) [22,23]. The FERM domain comprises three subdomains, F1, F2, and F3, and binds with cell membrane lipids, transmembrane proteins, and other membrane-associated proteins. The C-ERMAD domain contains a binding site interconnecting with filamentous actin (F-actin). A linker region rich in proline lies between the α-helical and C-ERMAD domains [24] (Figure 1A).

Like other ERM proteins, Ezrin can rapidly interconvert from an inactive closed to an active open conformation. In its closed conformation, Ezrin is localized in the cytoplasm where the FERM and C-ERMAD domains bind to each other, masking the F-actin and membrane-binding sites [25]. Ezrin becomes activated through a two-step process [26]. In the first step, Ezrin is recruited to the plasma membrane regions rich in phosphatidylinositol 4,5-bisphosphate (PIP_2_), where PIP_2_ binds to the FERM domain and exposes the conserved C-terminal threonine residue (Thr567). At the second step, several kinases (e.g., Rho Kinase, PKCα, PKCθ, NIK, Mst4 and LOK) can phosphorylate the Thr567 of Ezrin, causing Ezrin’s intramolecular head-to-tail interaction to be disrupted and Ezrin to subsequently become activated. Phosphorylated Ezrin is now able to bind to membrane-associated proteins and the actin cytoskeleton, acting as a membrane–cytoskeleton linker [12] (Figure 1B).

Ezrin regulates a diverse range of physiological processes. However, Ezrin dysfunction has been correlated with the progression of many diseases, including cancer and tumor metastasis [27]. Metastasis is the process in which tumor cells migrate from their primary site to colonize distant organs, which causes an overwhelming majority of cancer-related deaths [28]. The metastatic process can be described as the outcome of the interactions between tumor cells and the tumor microenvironment in which tumor cells undergo an evolutionary process to adapt and escape immunosurveillance [29]. Selective pressures of the tumor microenvironment and genetic alterations, such as oncogenic mutations and inactivation of tumor-suppressor genes, can select for tumor cells with the capability to grow despite the presence of environmental stressors, such as lack of oxygen and nutrients, low pH, reactive oxygen species, and the inflammatory response. The acquisition of these functions can facilitate the initiation and progression of metastasis, where tumor cells develop local advantages in the primary tumor microenvironment as well as selective advantages during the adaptation and takeover of a distant organ microenvironment. There is growing evidence that metastasis is a multidirectional process where tumor cells can seed both at distant sites and the primary tumor itself [30]. In this self-seeding metastasis model, cancer cells released from the primary tumor and distant places can re-enter the primary tumor mass, resulting in the primary tumor becoming a conglomerate of contiguous masses. This behavior may explain the aberrant motility of tumor cells, the ability of stem-like cells to initiate tumors, and the stroma’s and tumor’s interaction in metastasis. Metastasis has also been likened to an ecological dispersal, where tumor cells disseminate, similar to diasporas in which people disperse from an established homeland [31], suggesting that a successful metastasis requires the factors, such as an eligible primary tumor microenvironment, cancer cell migrants’ fitness, bidirectional ability to migrate between cancer sites, and suitable metastatic microenvironment sites. Cancers and metastases can be described as ecological diseases [32]; foundational ecological principles such as intraspecific (e.g., communication) and interspecific (e.g., competition, predation, parasitism, and mutualism) relationships could help elucidate tumor progression and the context of tumor metastasis. Here, we review the roles of Ezrin and its interacting proteins that ultimately allow it to be a key player in metastasis and aim to increase our understanding of how Ezrin contributes to metastatic disease and its potential as a therapeutic target.

## 2. Ezrin as a Linker between the Plasma Membrane and Cytoskeleton

**Figure 2:**
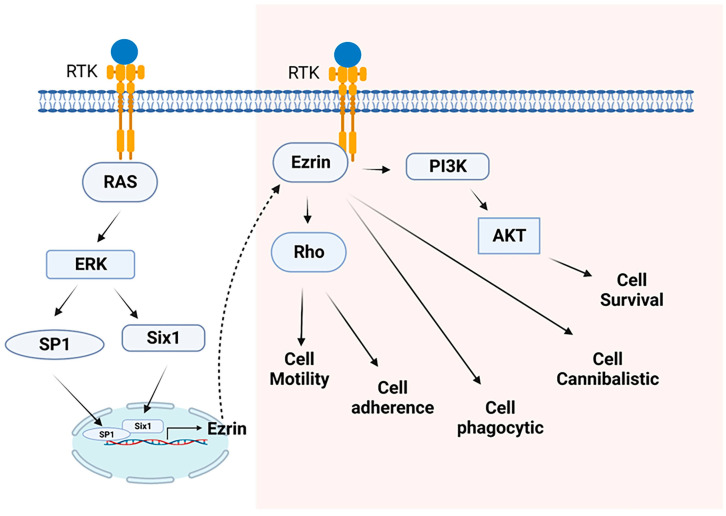
Ezrin controls several major cellular functions through the regulation of downstream signaling transduction pathways. Ezrin is transcriptionally regulated by several transcriptional factors, such as SP1 and Six1. Ezrin can activate Rho signaling to augment cell motility and cell adherence. Ezrin can also activate the PI3K/AKT signaling pathway to enhance cell survival. Moreover, Ezrin plays a role in cell phagocytic and cannibalistic processes.

Ezrin is a prime bridging protein capable of modulating several membrane–cytoskeletal interactions (MCIs), impacting cell behavior and regulating several essential cell functions (Figure 2).

### 2.1. Modulation of Membrane–Cytoskeleton Interactions

MCIs are necessary interactions between the plasma membrane and underlying actin cytoskeleton to communicate changes in the outside environment, serving as a hub for transmitting extracellular signals into the cell [33]. As a scaffold, Ezrin regulates several signal transduction pathways, such as PI3K signaling, Hepatocyte Growth Factor (HGF)/Met signaling, and RhoA signaling, impacting physiological function [20,34,35,36]. Ezrin is critical for survival as Ezrin-deficient mice were found to only survive 1.5 weeks after birth due to an intestinal defect preventing nutrient absorption [37,38].

Ezrin’s interactions with various proteins can modulate membrane–cytoskeleton interactions and result in differing outcomes. When activated by RhoA/ROCK1, Ezrin binds to Orai1 (oral calcium release-activated calcium modulator 1) to inhibit calcium entry in retracting blebs, resulting in a decrease in cytoplasmic calcium while also triggering the rapid assembly of the actin cortex [39]. While Ezrin is acetylated by lysine acetyltransferase PCAF (p300/CBP-associated factor), this prevents the phosphorylation of Ezrin at Thr567 and induces the translocation of Ezrin to the cytoplasm from the plasma membrane, promoting MDA-MB-231 breast cancer cell motility during migration and invasion [40]. When PRL3 (phosphatase of regenerating liver 3) dephosphorylates Ezrin at Thr567, this can initiate protrusion formation, inducing lamellipodia formation in osteosarcoma U2OS cells [41]. Additionally, Ezrin can interconnect with different types of membranes/substructures selectively; by interacting with curvature-sensing I-BAR (Inverse/Bin/amphiphysin/Rvs) domain proteins, Ezrin links to negatively curved membrane protrusions; when phosphorylating at Thr576, Ezrin connects to positively curved membrane protrusions [42]. Thus, Ezrin’s membrane–cytoskeleton modulatory functions are crucial for many essential cell functions, such as cell morphology, cell motility, and cell–cell adhesion, which will be discussed further in this section.

### 2.2. Maintenance of Cell Shape and Cell Structure

Ezrin maintains cell shape and structure. Several studies have demonstrated that Ezrin is important in maintaining a cell surface’s topography and associates with many cell surface structures on various cell types [17,43,44]. Ezrin deficiency in mice resulted in drastic changes in the morphology of microvilli, which become malformed and shortened [37,45].

Furthermore, Ezrin’s role as a crosslinker between the cortical actin network and the plasma membrane can affect membrane tension. In zebrafish mesodermal cells, researchers used either a nonphosphorylatable form of Ezrin or morpholinos that inactivated ERM protein function and found that membrane tension, adhesion energy, and dynamic tether force were significantly reduced in affected cells [46]. In contrast, modified super-active Ezrin expression in transgenic mice increased the membrane tension to 70% [47]. Later research found that modified plasma membrane interactions were associated with the reduced effectiveness of Ezrin [48]. The former was accomplished by microinjecting PIP_2_ to the inner leaflet of the lipid bilayer, which was previously shown to recruit more Ezrin to this area [49]. The latter was accomplished by injecting neomycin to mask PIP_2_, pharmacological inhibition of the phosphorylation of Ezrin using NSC668394, and short-interference RNA (siRNA) to reduce the amount of Ezrin. The researchers found that reducing the effectiveness of Ezrin crosslinking to the plasma membrane resulted in a lowered tether force, highlighting Ezrin’s central role in maintaining membrane tension [48]. Ezrin was also reported to maintain various cell surface configurations, including wrinkles and microridges [50]. Ezrin depletion could change the morphology of 16HBE human bronchial epithelial cells, resulting in a rounded appearance instead of the typical cobblestone pattern with many filopodia [51].

During phagocytosis, cells change their shape through many cycles of protrusion and retraction [52]. Ezrin, which is usually abundant at the periphery of neutrophils, was found to be completely absent from the phagocytic cup and pseudopodia, which are formed during phagocytosis [53,54]. This localized loss of Ezrin would be expected to decrease membrane tension locally [49], suggesting that localized pseudopodia formation is facilitated by the release of Ezrin from its crosslinking function [50].

A recent study found that Ezrin may initiate and directly regulate cell protrusions [41,55]. Reducing Ezrin’s affinity for actin with the inhibitor NSC668394 resulted in an increased frequency of cell protrusion; meanwhile, an Ezrin T567D mutant with an increased affinity for actin resulted in a decrease in the frequency of cell protrusion. The study also found that the level of Ezrin linked to actin is depleted before protrusion onset. When locally recruiting PLR3 to dephosphorylate Ezrin at Thr567, thereby reducing its affinity for actin, there was an increase in protrusion intensity.

### 2.3. Regulation of Cell–Cell Adhesion

Ezrin is also involved in the regulation of cell–cell adhesion. When Ezrin and other ERM proteins are suppressed, cell–matrix and cell adhesion are hampered in mouse epithelial cells [56]. In fact, Ezrin binds to cell adhesion proteins, e.g., CD44, CD43, intracellular adhesion molecule (ICAM)-1 and 2, and L-selectin, at the juxtamembrane amino acid sequences [15,57,58,59,60]. Therefore, directly crosslinking these cell adhesion proteins to actin filaments, such as when tethering CD44 to the cytoskeleton in macrophages, CD44 functions as a picket that forms a barrier to Fc receptor engagement in the plasma membrane [61]. L-selectin binds to Ezrin in leukocytes to induce leukocyte transendothelial migration; this interaction involves the recruitment of monocytes to endothelial monolayers [62]. Thus, Ezrin is crucial in delivering information between the actin cytoskeleton and adhesion proteins.

When dominant activated mutant Ezrin T567D was expressed in Madin–Darby canine kidney cells, it caused the extensive formation of lamellipodia and altered cell–cell contacts [16]; meanwhile, E-cadherin accumulated in intracellular compartments and decreased at the plasma membrane, suggesting that Ezrin is involved in cell–cell adhesion through trafficking E-cadherin to the plasma membrane. Additionally, the knockdown of Ezrin in THP-1 macrophages reduced binding to a confluent monolayer of human umbilical vein cells (HUVECs) by 75% by disrupting the interaction of Ezrin and cell adhesion molecular CD11b [63].

### 2.4. Regulation of Cell Movement

Ezrin regulates cell movement, thereby affecting cell motility and migration. HGF can induce the dissociation, migration, and remodeling of epithelial monolayers by modifying cell–cell adhesion and the actin cytoskeleton [20]. Crepaldi et al. found that Ezrin is crucial to this HGF-mediated morphogenesis in LLC-PK1 cells. When Ezrin is overproduced in LLC-PK1 cells, cell migration and tubulogenesis are enhanced. When a truncated variant of Ezrin is introduced, the morphogenic and mitogenic response to HGF is impaired instead. Protein kinase C (PKC) has been implicated in the promotion of cell migratory phenotypes [64], and when overexpressed in MCF-10A human breast cells, cell motility is enhanced [65]. This PKC-driven migratory response is directly correlated with Thr567 phosphorylation of Ezrin, and PKCα can form a molecular complex with Ezrin and hyperphosphorylate it at Thr567 [21]. Zhang et al. [66] utilized a mutant Ezrin T567D to find that constitutively activated Ezrin is localized at the cell rear and enhances cell migration.

Epithelial–mesenchymal transition (EMT) is a process of polar epithelial cells that normally interact with the basement membrane through their basal surface, lose their polarity, and undergo changes to become a mesenchymal cell type, which enhances their migratory capacity and invasiveness [67]. Ezrin also plays a significant role in this EMT conversion. Fröse et al. [68] found that EMT could promote podocalyxin (PODXL) to mediate the extravasation of breast and pancreatic carcinoma cells through direct interaction with Ezrin at its intracellular domain. Specifically, breast cancer cells use a PODXL–Ezrin signaling axis to rearrange the cytoskeleton and create a dorsal cortical polarity, changing cancer cells from a non-polarized rounded morphology to an invasive extravasation shape.

Altogether, a multitude of studies have demonstrated Ezrin’s critical role in many cellular functions. When Ezrin is dysregulated, cell morphology, cell–cell adhesion, and cell migration are impacted which can lead to further changes.

## 3. Ezrin Interacts with Metastasis-Related Proteins

**Figure 3:**
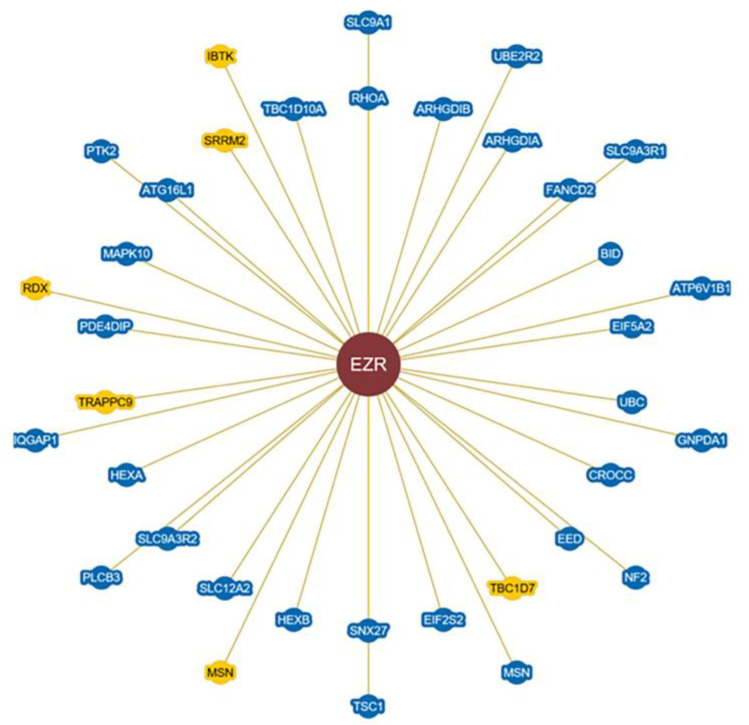
Representation of potential proteins interacting with Ezrin contained in the BioGRID database. Ezrin can interact with many proteins directly or indirectly, binding to proteins such as the Rho family, MSN, RDX, NF2, etc., to regulate cell adhesion, migration, and movement, as well as tumor metastasis. https://thebiogrid.org/204522/summary/mus-musculus/ezr.html, https://thebiogrid.org/113271/summary/homo-sapiens/ezr.html and http://www.hprd.org/summary?hprd_id=00475&isoform_id=00475_1&isoform_name=Isoform_1 (accessed on 18 May 2023).

Ezrin binds with many different metastasis-related proteins and induces a variety of reactions in tumor metastasis. In this section, we review the potential Ezrin-binding proteins and their functions (Figure 3).

### 3.1. ACTB

β-actin, encoded by the *ACTB* gene, is a cytoskeletal structural protein involved in cell growth, migration, and metastasis [69,70]. Ezrin interacts specifically with β-actin filaments and colocalizes within distal reaches of forward protrusions [71].

### 3.2. ADORA2B

*ADORA2B* encodes a G-protein-coupled adenosine receptor adenosine A2b receptor (A2bR), which can induce cAMP production [72]. Its overexpression has been correlated with tumor progression, which is important in immunosuppressive activity, tumor angiogenesis, and metastasis [73]. Upon agonist stimulation, A2bR is recruited to the plasma membrane, where it interacts with Ezrin, NHERF2, and PKA [74]. This interaction helps anchor A2bR to the plasma membrane and stabilize the receptor.

### 3.3. ADRA1B

*ADRA1B* encodes α1B-adrenergic receptor (α1B-AR), which is a G-protein-coupled receptor that activates mitogenic responses and regulates cell growth and proliferation in many cells. When α1B-AR is overexpressed and activated, it can induce neoplastic transformation and function as an oncogene [75]. Ezrin directly interacts with α1B-AR through a polyarginine motif on the receptor’s C-tail [76], regulating α1B-AR recycling to the plasma membrane, implying that Ezrin has a broader role in GPCR trafficking to promote tumor progression.

### 3.4. ARF6

ADP-ribosylation factor 6 (Arf6) is a member of the ADP-ribosylation factor family of small GTPases. Arf6 mainly functions at the plasma membrane, regulating endocytic pathways, protein trafficking and recycling, and actin remodeling at the leading edge of migrating cells [77]. A GDP-locked mutant of Arf6 (Arf6-T44N) has been found to localize with Ezrin in actin- and PIP_2_-enriched regions, suggesting that the Arf6 GDP-GTP cycle occurs at the plasma membrane [78]. Arf6 function is primarily dictated by the lifetime of its GTP-bound active form, which is orchestrated by the Arf6-specific GTPase-activating protein, ACAP4. A protein complex of Ezrin-ACAP4-Arf6 was found essential for volatile membrane remodeling [79]. Ezrin interacts directly with ACAP4 and colocalizes at the apical plasma membrane upon histamine stimulation [80]. Disrupting the Ezrin–ACAP4 interaction blocked apical membrane cytoskeleton remodeling during H^+^/K^+^ ATPase translocation to the apical membrane, suggesting that this interaction is linked to polarized epithelial cell secretion.

### 3.5. ARHGDIA

*ARHGDIA* encodes the protein of Rho guanine nucleotide dissociation inhibitory factors 1 (RhoGDI1). Part of the family of Rho GDP-Dissociation Inhibitors (Rho-GDIs), RhoGDI1 is an inhibitory regulator that forms a complex with the GDP-bound inactive form of Rho-GTPases, thereby inhibiting their activation [81]. Ezrin and Rho-GDIs directly interact at the FERM domain, which initiates the activation of Rho GTPases by reducing Rho-GDI activity and rescuing Rho GTPase from the Rho-GDP/GDI complex [82]. The interaction between Rho-GDIs and Ezrin can regulate the reorganization of actin filaments to drive cell shape, motility, and migration [81,83].

### 3.6. ARHGDIB

*ARHGDIB* encodes Rho guanine nucleotide dissociation inhibitory factors 2 (RhoGDI2), also known as LyGDI. Another member of the Rho-GDIs, LyGDI was found to function in cancer metastasis by anchoring Rho proteins to the cell membrane [84]. The expression of a C-terminal-truncated form of LyGDI (ΔC-LyGDI) induced pulmonary metastasis in 1-1ras1000 cells; in contrast, full-length LyGDI did not induce metastasis. Expressed ΔC-LyGDI was found primarily localized in the cell membrane and associated with the Rho family and ERM proteins, suggesting that LyGDI functions to anchor Rho family proteins to ERM proteins, allowing the spatial regulation of Rho family GTPase signaling during effector activation.

### 3.7. CD44

CD44 (the cluster of differentiation 44) is a cell-surface glycoprotein involved in cell–cell interactions and cell adhesion and migration. Ezrin is an intracellular anchor to CD44, linking it to the cytoskeleton [85]. CD44 binds directly to the FERM domain of Ezrin, and this interaction is regulated by Rho and PIP_2_ [58]. A recent study found that the binding efficiency of CD44 and FERM is directly impacted by a PIP2-dependent conformational switching of phosphorylated CD44 [86]. CD44 has been shown to promote tumor growth and invasiveness by recruiting Ezrin to its cytoplasmic tail and thus producing links to the cytoskeleton [87]. Interestingly, CD44 can also act as a tumor suppressor under confluent growth conditions, where binding with hyaluronate leads to activating and binding with Merlin, subsequently conferring growth arrest and contact inhibition. Osteopontin is reported to be an integral part of this hyaluronan–CD44–Ezrin complex, where the malignant secretion of osteopontin and CD44 variant isoforms has caused the migration of tumor cells to specific sites of metastasis formation [88,89].

### 3.8. CDH1

*CDH1* encodes E-cadherin, also known as Cadherin-1 or CD324. E-cadherin plays a major role in epithelial cell–cell adhesion. E-cadherin loss of function is associated with the disaggregation of tumor cells, thereby promoting their invasive and metastatic potential [90]. Ezrin interacts with E-cadherin to regulate cell–cell and cell–matrix adhesion, therefore controlling tumor cell adhesion and invasiveness [91]. In esophageal squamous cell carcinoma (ESCC), Ezrin activation and E-cadherin absence were observed to contribute to tumorigenesis and metastasis, where Ezrin and E-cadherin expression is negatively associated [92]. A similar finding in breast cancer showed that high Ezrin and low E-cadherin expression were more related to lymph node metastasis and poor prognosis [93]. In contrast, in intrahepatic cholangiocarcinoma, lack of or low Ezrin expression is associated with ectopic expression of E-cadherin and may coincide with the activation of an EMT-like process [94].

### 3.9. CLIC5

Chloride intracellular channel 5 (CLIC5) is a protein encoded by the *CLIC5* gene. CLIC5 was initially discovered as part of a protein complex from extracts of human placental microvilli alongside several actin-associated proteins such as Ezrin [95]. In renal glomerular podocytes, CLIC5 localizes to the apical plasma membrane of foot processes as a component of the Ezrin/NHERF2/podocalyxin complex and is required for podocyte structure and function. Deficient CLIC5 in mice markedly reduced Ezrin levels and increased susceptibility to glomerular injury, highlighting the importance of CLIC5 and Ezrin in podocyte integrity [96,97]. The mechanism of CLIC5 action involves interaction between PIP_2_-generating kinases and CLIC5 that causes clustered PIP_2_ to accumulate and subsequently facilitate Ezrin activation and cell surface remodeling [98]. CLIC5, Ezrin, and podocalyxin were overexpressed in hepatocellular carcinoma (HCC) and the inhibition of CLIC5 and podocalyxin resulted in decreased migration and invasion [99].

### 3.10. CTNNB1

*CTNNB1* encodes β-catenin, which regulates cell–cell adhesion and gene transcription. Like E-cadherin, Ezrin can regulate cell–cell and cell–matrix adhesion by interacting with β-catenin [91]. Part of the Wnt/β-catenin signaling pathway that determines normal tissue homeostasis, aberrant activation of β-catenin–T-cell factor (TCF) is a hallmark of colorectal cancer [100]. β-catenin is known to mediate tumor metastasis through interactions with Ezrin and the NF-κB pathway [101].

### 3.11. EGFR

EGFR (epidermal growth factor receptor) regulates epithelial cell growth and is overexpressed in various metastatic tumors [102]. Upon cell contact, ectopic apical Ezrin can increase cortical cytoskeleton contractility and EGFR internalization [103]. In non-small cell lung cancer (NSCLC) cells, Ezrin was found to enhance EGFR signaling and regulate EGFR trafficking to the nucleus. When Ezrin expression is inhibited, both EGF-induced phosphorylation and nuclear translocation of EGFR are reduced. Additionally, the phosphorylation of Ezrin at Tyr146 resulted in increased proliferation, colony formation, and erlotinib drug resistance [104].

### 3.12. FAS

Fas, also known as CD95, apoptosis antigen 1 (APO-1), or tumor necrosis factor receptor superfamily member 6 (TNFRSF6), is a death receptor encoded by the *FAS* gene. Fas can trigger apoptosis in various cell types, where Fas activation can lead to the formation of a death-inducing signaling complex [105]. Human T lymphocytes undergo cell membrane polarization through an Ezrin-mediated interaction with the actin cytoskeleton, where Ezrin and Fas both colocalize at the polarization site. This mechanism renders T lymphocytes susceptible to Fas-mediated apoptosis [106], therefore negatively regulating the T cell function and promoting tumor metastasis [107].

### 3.13. ICAM1, ICAM2, and ICAM3

ICAM-1, ICAM-2, and ICAM-3, intercellular adhesion molecules 1, -2, and -3, are cell surface glycoproteins encoded by the *ICAM1*, *ICAM2*, and *ICAM3* genes, respectively. ICAMs mediate binding to leukocyte β_2_ integrins (CD11/CD18) such as LFA1 and Mac1 during inflammation and immune response [108]. Ezrin is directly involved in ICAM-2 subcellular distribution and adhesive function, where Ezrin can trigger the redistribution and accumulation of ICAM-2 in uropods [109]. PIP_2_ can enhance Ezrin and ICAM-2 interaction. ICAM-3, in contrast, did not bind with Ezrin despite the presence of PIP_2_ [59]. In polarized T lymphocytes, however, Moesin interacts with ICAM-3 and both are redistributed to the uropod upon T lymphocyte simulation [110]. These cells exhibit low ICAM-1 function and with ICAM and ERM proteins having overlapped and redundant functions, they may display varying combinations depending on the cellular environment [59]. A recent study reported that the interaction of ICAM-1 and Ezrin destructs the endothelial barrier and enhances vascular permeability, therefore promoting tumor metastasis [111].

### 3.14. IQGAP1

IQ motif containing GTPase activating protein 1 (IQGAP1) is a scaffolding protein encoded by the *IQGAP1* gene and functions in metastatic signaling pathways by recruiting signaling intermediates for efficient signal transduction [112,113]. Ezrin interacts with IQGAP1 at the FERM domain, forming a hub for concentrating signaling complexes. Both Ezrin and IQGAP1 colocalize in the submembranous cytoskeleton and cellular protrusions of human epithelial cells, and when Ezrin is knocked down, the cortical localization of IQGAP1 is reduced [114]. In line with this, another study established that IQGAP1 is positioned at the cell cortex by Ezrin [115]. Thus, Ezrin’s involvement in recruiting IQGAP1 to the cell cortex establishes a tight spatial control of actin dynamics by allowing regulators of actin organization, such as Cdc42 and Rac1, to be placed in proximity [114].

### 3.15. L1CAM

L1CAM (L1 cell adhesion molecule) is a glycoprotein encoded by the *L1CAM* gene and is involved in cell adhesion and migration. L1CAM expression is frequently upregulated in cancer patients and has a distinct role in the metastatic cascade, promoting dissemination, colonization, and metastatic growth [116]. In ESCC, L1CAM was found to upregulate the expression of Ezrin through activation of integrin β1/MAPK/ERK/AP1 signaling and promoted tumorigenicity [117]. Additionally, Ezrin and L1CAM binding was found to be required for L1CAM-mediated metastasis through the NF-κB signaling pathway in CRC [101]. Ezrin and L1CAM were found to interact during EMT [118] and are important prognostic melanoma markers associated with hypoxia, which can affect EMT and may be responsible for early metastatic dissemination [119]. Interestingly, interrupting the binding of Ezrin and L1CAM suppressed the metastases in CRC [101].

### 3.16. MSN

The *MSN* gene encodes Moesin (Membrane-Organizing Extension Spike Protein), part of the ERM family of proteins that link the plasma membrane with actin filaments. Moesin is highly expressed in the lungs, spleen, kidneys, and endothelial cells [43]. Its phosphorylation at Thr558 activates Moesin to its open conformation. While sharing many properties similar to Ezrin and Radixin, the three family members have distinct and overlapping distribution patterns that coincide with their functions and roles. Ezrin and Moesin are predominantly expressed in lymphocytes and have unique and redundant functions in lymphocyte adhesion, activation, and migration [120,121].

### 3.17. NF2

The neurofibromatosis type 2 gene (*NF2*) encodes a tumor suppressor of Moesin–Ezrin–Radixin-Like Protein (Merlin). A member of the FERM domain-containing 4.1 superfamilies, Merlin shares an evolutionarily conserved domain and sequence homology with ERM proteins but lacks the actin-binding site on the C-ERMAD domain, instead having a unique actin-binding motif in the FERM domain [122]. Merlin plays a key role in contact inhibition of cell proliferation and signal transduction, regulating pathways such as PI3K/AKT, Raf/MEK/ERK, and mTOR [123]. When a mutation in the *NF2* gene occurs, tumorigenesis can occur as its tumor-suppressing function ceases [124,125], while a splicing form of Merlin promotes hepatocellular carcinoma metastasis [126]. Zhou et al. [127] found that wildtype Merlin and Ezrin overexpression in human pancreatic cancer cell line SW1990 inhibited cell proliferation, migration, and adhesion. In contrast, the overexpression of T567D Ezrin promoted these migratory behaviors, suggesting that Ezrin can inactivate NF2 tumor suppressor and promote metastasis [128].

### 3.18. PALLD

*PALLD* encodes palladin, a component of actin-containing microfilaments that control cell shape and adhesion. Part of the myotilin/myopalladin/palladin family, palladin is primarily expressed in smooth muscle and nonmuscle, localizing along actin microfilaments in a periodic manner typical for components of dense bodies of smooth muscle in stress fibers [129]. Palladin has been identified as an Ezrin-associated protein that regulates the microfilament localization of Ezrin. Palladin contains three Ig-domains, and Ig-domains 2-3 bind to the active form of Ezrin. The existence of an Ezrin/palladin complex unites the Rho-pathway and VASP-mediated control of the acto-myosin system. This interaction can significantly affect a tumor’s metastatic capability [130].

### 3.19. PRCKA

Protein kinase C alpha (PKCα) is an enzyme encoded by the *PRCKA* gene. PKCα has been implicated in promoting β1 integrin-mediated cell migration as part of the protein kinase C family. PKCα overexpression is associated with increased cell motility and invasive potential [64]. As stated, PKCα forms a molecular complex with Ezrin, which is hyperphosphorylated at Thr567, activating Ezrin and driving the migratory response [21]. In breast cancer cells, Ezrin upregulation correlates with HER2 expression. The inhibition of Ezrin blocked HER2 signaling and caused a PKCα-mediated internalization and degradation of HER2, inhibiting proliferation and promoting apoptosis in HER2-positive breast cancer cells [131].

### 3.20. PTK2

Protein tyrosine kinase 2 (PTK2), also known as focal adhesion kinase (FAK), is an enzyme encoded by the *PTK2* gene. FAK is important in various cell functions, including motility, adhesion, metastasis, invasion, survival, apoptosis, and angiogenesis [132]. It has also been found to play an important role in EMT, cancer stem cells, and tumor microenvironments [133]. FAK has been shown to activate the PI3K/AKT/mTOR pathway, which can support tumor cell survival [134]. Ezrin interacts with FAK and induces its activation, thus acting as a regulator of the FAK/PI3K/AKT pathway [135]. In pancreatic cancer, Ezrin was shown to accelerate cancer proliferation and metastasis by activating the FAK/AKT signaling pathway and upregulating FAK expression [136].

### 3.21. RDX

The *RDX* gene encodes Radixin, part of the ERM family of proteins that link the plasma membrane with actin filaments. Radixin is highly expressed in the liver, and phosphorylation at Thr564 activates Radixin to its open conformation. Like Ezrin and Moesin, Radixin is overexpressed in many tumor tissues [137] and can modulate the viral infection process for several viruses by regulating stable microtubule function [138]. The knockdown of Radixin by shRNA in glioblastoma U251 cells was found to significantly inhibit tumor growth and upregulate thrombospondin-1 (TSP-1) and E-cadherin while downregulating MMP9 [139]. In gastric carcinoma SGC-7901 cells, the inhibition of Radixin significantly suppressed cell migration and invasion but enhanced adhesion by upregulating E-cadherin via the NF-κB/snail pathway [140]. Additionally, microRNA-200b, associated with the development of multiple tumors, was found to regulate breast cancer cell proliferation by targeting Radixin expression [141]. While the precise molecular function of Radixin remains elusive, its implication in these pathways may suggest a unique role in metastasis.

### 3.22. RHOA

RhoA (Ras homolog family member A), a protein encoded by the *RHOA* gene, is part of the Ras superfamily of small guanosine triphosphatases (GTPases). RhoA plays a critical role in signal transduction, regulating cell morphology, growth, movement, and the cell cycle by switching between its inactive GDP-bound form to its active GTP-bound form [81]. Ezrin interacts with RhoGDI by dissociating it from RhoA, thus allowing RhoA to activate. Podocalyxin, a major protein in podocytes, has been shown to associate with Ezrin in recruiting RhoGDI to activate RhoA and actin reorganization [142]. RhoA has also been shown to regulate breast cancer metastasis as an upstream signaling factor of Ezrin, where increasing RhoA phosphorylation resulted in enhanced Ezrin expression [143,144]. In contrast, inhibiting RhoA phosphorylation significantly suppressed Ezrin expression. Additionally, RhoA expression has been correlated with Ezrin expression in osteosarcoma, where Ezrin expression may modulate RhoA expression [145]. In ovarian cancer, Ezrin can positively regulate active forms of RhoA, promoting stress fiber formation, cell invasion and metastasis [146].

### 3.23. ROCK1

Rho-associated, coiled-coil-containing protein kinase 1 (ROCK1) is a serine/threonine protein kinase that plays a significant role in the actomyosin cytoskeleton and is encoded by *ROCK1*. ROCK1 is a major downstream effector of RhoA and plays a large role in cell motility, metastasis, and angiogenesis [147]. ROCK1 has been shown to phosphorylate Ezrin at Thr567 [148]. Interestingly, Ezrin can act upstream and downstream of ROCK1 in the RhoA/ROCK signaling pathway, where ROCK1 activates Ezrin, dissociates RhoGDI from RhoA, and subsequently activates RhoA/ROCK [149]. In Jurkat cells, Fas-mediated apoptosis is regulated by the Rho/ROCK-dependent phosphorylation of Ezrin and Moesin, where Rho or ROCK inhibition prevents their phosphorylation, disrupting the formation of Fas aggregates and subsequent apoptosis induction [150].

### 3.24. S100P

S100 calcium-binding protein P (S100P) is an EF-hand protein encoded by the *S100P* gene. S100 proteins are involved in the regulation of membrane–cytoskeleton interactions and cytoskeleton dynamics. Dimeric S100P was found to bind to and activate dormant Ezrin in a Ca^2+^-dependent manner. S100P can also unmask the F-actin binding site on Ezrin, partially activating Ezrin in response to Ca^2+^ stimulation [151]. This interaction between Ezrin and S100P has been implicated in tumor cell migration, where the resulting activation of Ezrin promotes the transendothelial migration of tumor cells. With their overexpression in highly metastatic cancers, direct interaction between these proteins and S100P-mediated Ezrin activation may have a more prominent prometastatic role [152].

### 3.25. SCYL3

*SCYL3* encodes the protein PACE-1 (protein-associating with the carboxyl-terminal domain of Ezrin). Initially characterized in human breast cancer cells, PACE-1 binds to the C-terminal domain of Ezrin and colocalizes with Ezrin in the lamellipodia. It may regulate cell adhesion/migration complexes and could function as a scaffold protein to bring kinase activity near Ezrin [153].

### 3.26. SDC2

Syndecan-2, a cell surface heparan sulfate proteoglycan encoded by the *SDC2* gene, mediates cell–cell and cell–matrix adhesion and cytoskeletal organization. The N-terminal domain of Ezrin binds to syndecan-2 in a dose-dependent manner, where syndecan-2 contains a specific and unique Ezrin-binding sequence, suggesting a distinct regulation [154]. In fibrosarcoma, IGF-I is an anabolic growth factor that can promote tumorigenesis by inhibiting apoptosis and promoting cell cycle progression. IGF-I enhanced Ezrin phosphorylation levels, SDC-2 expression, and the formation of an SDC-2 and Ezrin complex. It was also revealed that SDC-2 colocalizes to the IGF-I receptor for recruiting Ezrin in the plasma membrane to enhance actin polymerization, ultimately facilitating IGF-I-dependent fibrosarcoma cell migration [155].

### 3.27. SELL and SELP

Selectin L (CD62L) and Selectin P (CD62P), encoded by the *SELL* and *SELP* genes, are transmembrane proteins functioning as cell adhesion molecules. Selectin L contains an ERM binding domain; mutants with defective ERM binding have decreased microvilli localization and reduced tethering to Selectin P glycoprotein ligand-1 (PSGL-1), suggesting an important role for ERM proteins in Selectin L function [156]. Additionally, the uropods of T lymphocytes are enriched in ERM proteins, PSGL-1, and flotillins. It was found that the expression of T567D Ezrin can induce cell polarization and the formation of uropods enriched in PSGL-1 and flotillins, suggesting ERM involvement in the negative regulation of T lymphocytes [157,158].

### 3.28. SLC9A1

*SLC9A1* (solute carrier family 9, member A1) encodes Na^+^/H^+^ exchanger 1 (NHE1), a membrane protein that transports Na+ into the cell and H^+^ out of the cell. Localized in invadopodia, the upregulation of NHE1 has been correlated with tumor malignancy [159,160,161,162]. NHE1 acts as a scaffold protein and interacts with numerous proteins through its regulatory C-terminus [163]. At the C-terminal tail, NHE1 contains two ERM protein-binding motifs, and this interaction regulates many important cellular events such as cell migration, signaling complexes, and resistance to apoptosis [164,165,166]. When ERM and NHE1 are associated, apoptosis is stalled through the activation of Akt3 and NHE1 becomes localized in the lamellipodia of migrating cells [167]. A recent study demonstrated that elevated extracellular fluid viscosity promotes cell migration and cancer dissemination [168]. The high viscosity imposes a mechanical load that induces the formation of a denser actin network where Ezrin enrichment promotes NHE1 polarization and, subsequently, NHE1-dependent cell swelling.

### 3.29. SLC9A3R1

Na^+^/H^+^ exchanger 3 regulatory factor 1 (NHERF1) or ERM-binding phosphoprotein 50 (EBP50) is encoded by the *SLC9A3R1* (solute carrier family 9, member 3 regulator 1) gene. NHERF1/EBP50 is a PDZ-scaffold protein that significantly regulates the cancer signaling network by assembling cancer-related proteins [169]. Scaffold proteins coordinate specific signaling pathways by locally concentrating, compartmentalizing, and positioning transporters/receptors or enzymes in the vicinity of their substrates [170,171,172,173]. EBP50 can have either antitumor or pro-tumor functions, dictated by its expression or subcellular localization in either the plasma membrane or nucleus, and this dual function encompasses its regulation of several major signaling pathways, including receptor tyrosine kinases PDGFR and EGFR, the PI3K/PTEN/AKT pathway, and the Wnt/β-catenin pathway [169].

### 3.30. SLC9A3R2

*SLC9A3R2* (solute carrier family 9, member 3 regulator 2) encodes a PDZ-scaffold protein of Na^+^/H^+^ exchanger 3 regulatory factor 2 (NHERF2). NHERF2 shares about 52% amino acid identity with EBP50 and has the same PDZ domain structure [174]. Both EBP50 and NHERF2 play an essential role in the NHE3/Ezrin/cAMP-dependent protein kinase II signaling complex, a process required for ion transport inhibition through the phosphorylation of NHE3 [175,176]. EBP50, NHERF2, and ERM have a distinct cell-type-specific expression that parallels their binding preferences [177]. In pulmonary endothelial cells, NHERF2 was shown to be crucial in filopodia formation and endothelial cell migration by mediating the phosphorylation of ERM via ROCK2 [178]. In glomerular podocytes, Ezrin has also been reported to form a multi-protein complex with NHERF2 and podocalyxin [179], and the loss of Ezrin reduced susceptibility to glomerular injury in mice [180]. A recent study found that this protein complex also interacts with nephrin and ephrin-B1, forming an axis critical to podocyte injury [181].

### 3.31. SNX27

Sorting nexin 27 (SNX27) is a sorting nexin family member and plays a critical role in the endosomal recycling of many transmembrane receptors. SNX27 contains a Phox homology domain that binds to phosphatidylinositol phospholipids and regulates its localization to the endosome, as well as a PDZ (Psd-95/Dlg/ZO1) domain and an atypical FERM domain that both function to bind to cargo receptors containing a short NPxY sequence motif [182,183]. SNX27 interacted with the small GTPase Ras, which has been implicated in oncogenic signaling pathways [184]. The Ras interaction arises through the FERM F1 subdomain, suggesting that other FERM domain proteins share a similar binding activity [185]. SNX27 was also found to mediate cancer progression. The loss of SNX27 reduced tumor growth and proliferation in breast cancer cells and its ability to minimize aggressiveness and invasive capacity via modulation of EMT marker, Vimentin, and cell–cell junction markers, E-cadherin and Claudin-5 [186].

### 3.32. SPN

*SPN* encodes CD43 (also known as sialophorin or leukosialin), a sialoglycoprotein that plays an essential role in T lymphocyte activation, proliferation, apoptosis, and migration. It was found that both Ezrin and Moesin interact with CD43, regulating its redistribution to T lymphocyte uropods and inhibiting T cell and APC interaction [187]. This interaction can facilitate transendothelial migration and T lymphocyte recruitment [188]. Additionally, CD43 can be phosphorylated at Ser76 to regulate T cell trafficking through association with ERM proteins [189]. The overexpression of CD43, along with CD44 and ICAM-2, significantly induced microvillar elongation and ERM recruitment, indicating that these proteins function to organize microvilli for cortical morphogenesis [190].

### 3.33. TSC1

TSC1 (tuberous sclerosis-1) is a tumor suppressor protein encoded by the *TSC1* gene. TSC1 was found to regulate cell adhesion through its interaction with ERM proteins and Rho [191]. Interaction with Ezrin is required to activate Rho and inhibit TSC1 function in cells containing focal adhesions, resulting in loss of adhesion to the cell substrate. Additionally, both Ezrin and TSC1 were found to be required to activate the Dbl oncogene, a Rho guanine nucleotide exchange factor (GEF) [192]. The knockdown of Ezrin and TSC1 and expression of mutant TSC1 cannot bind with Ezrin, resulting in the inhibition of Dbl activity.

### 3.34. VCAM1

Vascular cell adhesion molecule-1 (VCAM-1) is a cell adhesion molecule encoded by the *VCAM1* gene. VCAM-1 interacts with Ezrin and Moesin during leukocyte adhesion and transendothelial migration, and all three colocalize at the apical surface of the endothelium. An endothelial docking structure forms from the clustering of VCAM-1, ICAM-1, and activated Ezrin and Moesin during leukocyte adhesion, anchoring and partially embracing the leukocyte [193]. Additionally, VCAM-1 expression in breast cancer cells is associated with lung relapse; cancer cells expressing VCAM-1 tether metastasis-associated macrophages and the resulting clustering of VCAM-1 binds with Ezrin and triggers the activation of the Akt survival pathway through juxtacrine activation. This can protect cancer cells from proapoptotic cytokines and allow for metastasis at leukocyte-rich sites, such as the lungs [194].

## 4. Role of Ezrin in Immunity to Prevent Immune Attack

**Figure 4:**
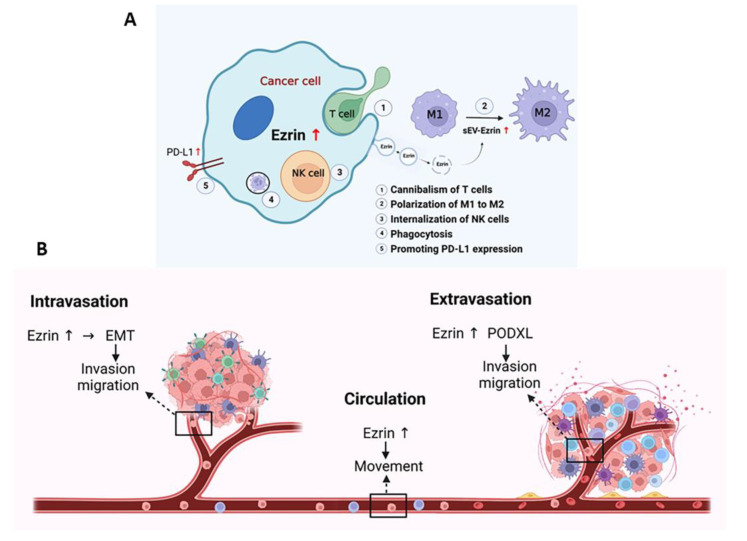
Ezrin is critical for metastasis and regulates multiple steps of the metastatic process in a variety of ways. (**A**) Ezrin expression in tumor cells promotes many interactions between tumor cells and the immune system, protecting metastatic tumor cells from immune cell attack. (**B**) Upregulated Ezrin in metastatic tumor cells helps regulate all steps of the metastatic cascade, including initial dissemination, circulation, seeding, colonization, and survival at distant organ sites. For example, Ezrin controls the EMT molecules to initiate tumor migration and invasion from the primary site in the intravasation step. Disseminating tumor cells enter the bloodstream and highly express Ezrin for tumor cells’ movement in circulation. The circulating tumor cells with higher Ezrin expression are more closely associated with distant metastasis. Ezrin and PODXL directly interact to rearrange the cytoskeleton to change cell morphology towards an invasive extravasation-competent shape. Lastly, Ezrin also promotes the survival of tumor cells at distant organs following extravasation.

Ezrin is primarily known for connecting membrane proteins to the actin cytoskeleton [14,195]. This process has been linked to the metastatic behavior of tumors, where adhesion molecules, through Ezrin-mediated linkages to actin, confer to tumor cells the capacity to migrate within tissues, through vessels, and attach to metastatic organs [89,196]. However, Ezrin has also been implicated in many interactions with the immune system that protect cells from immune attack (Figure 4A).

Tumor cells have been found to exhibit phagocyte-like behavior; studies have shown dead cells and undefined particles phagocytosed within various tumor cells [197,198]. Cell lines derived from metastatic tumors also exhibited vigorous phagocytic activity [196]. Interestingly, Ezrin has been found to have an essential role in the phagocytic process of macrophages [199]. Localized within phagocytic vacuoles, the downregulation of Ezrin leads to the decreased phagocytic activity of metastatic tumor cells [200]. Fais [196] had two proposals for this behavior: (i) tumor cells being able to phagocytose allows them to escape immune surveillance by subtracting material needed for processing antigens, or (ii) tumor cells can survive in adverse environments during tumor growth by using phagocytic processes to feed on apoptotic cells or ECM components. Further expanding on this, the phenomenon of cell cannibalism was explored. Cell cannibalism involves the engulfment of cells within other cells and has been observed in malignant tumors, including human metastatic melanoma cells [200]. Lugini et al. [201] found that live lymphocytes, specifically melanoma-specific CD8+ T cells, were cannibalized by metastatic melanoma cells, not primary melanoma cells. This cannibalistic activity significantly increased metastatic melanoma cell survival, particularly in unfavorable conditions. Incorporating an Ezrin deletion mutant resulted in a significantly reduced percentage of melanoma cells engulfing live lymphocytes. Additional data suggested that Ezrin may provide an altered connection between actin and caveolin-1-enriched vacuoles, the driving structure of the cannibalistic process.

Ezrin also interacts with other immune cells to promote metastasis and has been found to influence immune cell polarization, emigration, and intracellular adhesion. In pancreatic ductal adenocarcinoma (PDAC), PDAC-derived small extracellular vesical Ezrin (sEV-EZR) was found to regulate macrophage polarization and promote PDAC metastasis [202]. There are two types of macrophages: M1 macrophages, which are pro-inflammatory and have antitumor functions, and M2 macrophages, which are anti-inflammatory and have pro-tumor features [203]. It was found that PDAC-derived sEVs modulate the divergence of macrophages to the M2 phenotype. At the same time, PDAC-shEZR-derived sEVS polarized macrophages to the M1 phenotype and reduced the amount of liver metastasis [202]. A similar finding was also observed in THP-1 macrophages. Khan et al. [63] found that Ezrin skews the differentiation of THP-1 macrophages toward the pro-tumorigenic M2 phenotype, contributing to factors that stimulate tumor cell migration, invasion, and clonogenic growth, as well as the expression of mRNAs encoding vascular endothelial growth factor (VEGF)-A and matrix metalloproteinase (MMP)-9. Alongside regulating the expression of angiogenic factors in macrophages, Ezrin can also regulate their expression in tumor cells. These bidirectional responses can generate a positive feed-forward loop where tumor cell secretions induce myeloid cell activation and vice versa [63]. Ezrin also plays a role in T lymphocyte polarization and migration [204]. In response to adhesion or chemotactic stimuli, T cells polarize and display as two poles: a lamellipodium-like structure at the leading edge and a protrusion at the trailing edge called the uropod. Ezrin, through its control of plasma membrane tension and role in the generation of the uropod, is able to regulate the intracellular signaling in lymphocyte activation [205]. Additionally, Ezrin’s role in forming actin-based structures, such as the immune synapse in macrophages and the phagocytic cup, highlights its role in the effector immune response.

A recent study linked circulating actin-binding proteins with circulating tumor cells of laryngeal squamous cell carcinoma (LSCC) [206]. It was found that the blood serum of patients with LSCC had the highest levels of Ezrin. Ezrin was mainly expressed by circulating tumor cells in contrast to leukocytes, which expressed other actin-binding proteins. Ezrin’s expression in circulating tumor cells is likely related to the level of circulating Ezrin in the blood flow. While the role of circulating actin-binding proteins in cancer has been poorly studied, the correlations in actin-binding protein levels with circulating tumor cells and leukocytes may indicate the body’s immune response to tumor growth.

Another way Ezrin aids tumor cells in evading the immune system is through its interaction with programmed cell death ligand-1 (PD-L1). PD-L1 is an immune checkpoint protein that binds to programmed cell death-1 (PD-1) and is highly expressed on the cell surface of various cancers to abolish T-cell-mediated immunosurveillance [207]. The binding between PD-L1 and PD-1 leads to T cell dysfunction by inhibiting T cell activity and proliferation, facilitating T cell exhaustion and anergy, and inducing apoptosis of activated T cells [208]. In human adenocarcinoma cells (LS180), Ezrin was found to colocalize with PD-L1 in the plasma membrane and may function as a scaffold protein mediating the localization of PD-L1 to the plasma membrane [209]. Furthermore, the gene silencing of Ezrin resulted in substantially decreased PD-L1 cell surface expression without affecting its mRNA expression. A similar finding was observed in human cervical adenocarcinoma cells (HeLa) [210] and in human uterine endometrial cancer cells (HEC-151) [211].

NK cells are another immune cell with which Ezrin interacts. NK cells are key players in orchestrating immune responses and eliminating metastatic tumor cells [212]. However, the internalization of NK cells into their tumor cell targets can lead to their self-destruction. Wang et al. [213] found that Ezrin plays an important role in NK cell internalization, where the phosphorylation of Ezrin by PKA elevates this internalization process and leads to programmed cell-in-cell death. In contrast, suppression of Ezrin phosphorylation attenuates NK cell internalization into tumor cells. The internalization of NK cells may be a mechanism of tumor progression, similar to the aforementioned cannibalism of T cells. However, NK cell internalization is distinct from cannibalism since internalization is an active process that requires NK cell viability, while cannibalism is a phagocytic process where tumor cells can phagocytose both viable and dead cells. While it is unclear whether this process contributes to the malignant phenotype of a tumor, the hyperphosphorylation of Ezrin relating to tumor metastasis implicates the importance of Ezrin in tumorigenesis. Moesin also regulates NK cell homeostasis and survival, where Moesin-deficient NK cells exhibit increased cell death [214]. Since NK cells play a critical role in antitumor immunity, further understanding of these interactions with NK cells may lead to the identification of a promising new therapeutic target.

## 5. Ezrin as a Target for Treating Metastatic Disease

Ezrin has been widely studied as a possible therapeutic target for treating metastatic disease. This is due to its identification as a critical regulator of metastasis in several cancers [215], playing a role in nearly every step of the metastatic cascade (Figure 4).

To successfully metastasize, the tumor cell must make numerous adjustments and survive a series of challenges involving intravasation, blood or lymph system circulation, extravasation, and growth at distant organs [29]. In line with this, Ezrin has been implicated in many steps of the metastatic cascade, and many of these steps have been mentioned previously. Ezrin plays a large role in EMT, which initiates the escape of cancer cells from their primary site and enhances their migratory capacity and invasiveness [67]. Here, Ezrin can control the function of various EMT-associated transcription factors, such as Snail and Twist [216], and activate signaling pathways that facilitate the EMT process, such as the NF-κB pathway [217]. Following EMT, Ezrin can further enhance cancer cell invasion and migration by facilitating cytoskeleton remodeling [20], increasing cell motility [21], as well as polarizing macrophages towards the M2 type [63,202]. Following this, tumor cells undergo intravasation and circulate in the bloodstream. Ezrin is highly expressed by circulating tumor cells, and this has been associated with higher levels of Ezrin in the bloodstream in LSCC [206]. This has also been observed in osteosarcoma, where circulating tumor cells with higher Ezrin expression are associated with distant metastasis [218]. Afterward, cells undergo extravasation, moving out of the bloodstream to colonize the surrounding tissue. Ezrin and PODXL directly interact to rearrange the cytoskeleton to change cell morphology towards an invasive extravasation-competent shape [68]. Lastly, Ezrin also promotes the survival of tumor cells at distant organs following extravasation. In breast cancer, Ezrin was found to be required for initial seeding and colonization at distant organ sites, such as in the lungs [219]. Together, these findings highlight Ezrin’s important role in the metastatic process.

Much research has indicated that Ezrin overexpression is strongly associated with tumor metastasis and poor patient prognosis, acting as a key player in various cancer types. This has been observed in rhabdomyosarcoma (RMS) [215], breast cancer [220,221], PDAC [202], melanoma [36], ESCC [222], prostate cancer [223], and nasopharyngeal carcinoma [224], among others. Ezrin is abundantly expressed in highly metastatic cells in RMS compared to poorly metastatic cells [215]. Additionally, the homeoprotein transcription factor Six1, which has been shown to strongly influence the metastatic potential of RMS cells, transcriptionally activates Ezrin and is required to promote metastatic dissemination [225]. In ESCC cells, lysyl oxidase-like 2 (LOXL2) upregulates the phosphorylation of Ezrin to promote cytoskeletal reorganization and cell invasion [222]. In melanoma, aberrant HGF/Met signaling can promote melanoma metastasis by enhancing Ezrin expression via the Sp1 transcription factor [36].

Given this, Ezrin represents a promising therapeutic target for cancer patients. Many studies have demonstrated that the expression of a phosphorylation-deficient constitutively inactive Ezrin mutant (T567A) significantly reduced invasion and metastasis, in contrast to a phosphorylation-mimetic mutant (T567D) [66,215,225,226,227,228]. Thus, targeting the phosphorylation of Ezrin may best prevent cancer progression [229] (Table 1).

One approach is to target Ezrin directly with small-molecule inhibitors. NSC305787 and NSC668394 are small-molecule inhibitors that directly bind to Ezrin to prevent phosphorylation at Thr567, and therefore interactions between Ezrin and actin, and were found to inhibit osteosarcoma cell Ezrin-mediated motility [230]. NSC305787 can also reduce the incidence of lung metastasis of osteosarcomas [231]. Interestingly, MMV667492, a quinolone-based derivative, was found to have structural similarity to NSC305787 and exhibited more potent anti-Ezrin activity than NSC305787 [232]. Furthermore, the inhibition of Ezrin by NSC668394 can reduce the metastatic burden at the distal axillary lymph node and lungs in breast cancer [233]. In this same study, however, there were minimal changes to primary tumor growth, implying that a combination of therapies that target both the primary tumor and metastatic cell populations should be considered [236]. Lapatinib, a dual kinase inhibitor that targets HER2 and EGFR and is used to treat patients with HER2-positive breast cancer, was found to be more effective at promoting cell death and inhibiting proliferation when combined with NSC668394 [131]. It is important to note that certain epigenetic drugs, such as HDAC inhibitors (TSA) and demethylating agents (5-Aza), were able to stimulate metastasis through the enhanced expression of Ezrin and can even reactivate Ezrin expression in otherwise low-Ezrin cell lines [238]. This suggests that inhibiting Ezrin expression may be required for future epigenetic drugs to be successful.

## 6. Conclusions

Since its discovery in 1983, the knowledge and understanding of Ezrin’s biology and function have continuously expanded. Its role as a linker between the plasma membrane and cytoskeleton has been well studied, as many of its functions described here have contributed to its role in tumor metastasis. Modulating several of the membrane–cytoskeleton interactions, maintaining the cell shape and structure, and regulating cell–cell adhesion, as well as regulating cell movement, can confer to tumor cells the ability to survive and successfully metastasize. Additionally, its regulation by many signaling molecules through phosphorylation and conformational changes heavily dictates its function. Ezrin also has a host of binding partners that can explain how Ezrin is intertwined in various oncogenic signaling pathways. Furthermore, Ezrin’s interactions with the immune system allow it to aid tumor cells in evading immune surveillance. However, the molecular mechanisms of Ezrin’s specific interactions are very complex, and more understanding is needed to elucidate its exact pathophysiological roles. Ezrin has become a promising therapeutic target in reducing the metastatic burden of several cancer types. The rising potential of treatments involving Ezrin will continue to grow as new developments emerge and novel mechanisms of Ezrin are uncovered.

## Figures and Tables

**Table 1:** Inhibition of Ezrin for treating metastatic diseases.

Name	Format	Mechanism	Disease	Status	Reference(s)
NSC305787	small molecular inhibitor	inhibits Ezrin phosphorylation	osteosarcoma	pre-clinical	[230]
NSC668394	small molecular inhibitor	inhibits Ezrin phosphorylation	osteosarcoma	pre-clinical	[230,231]
MMV667492	small molecular inhibitor	inhibits Ezrin phosphorylation	osteosarcoma	pre-clinical	[232]
NSC668394	small molecular inhibitor	inhibits Ezrin phosphorylation	breast cancer	pre-clinical	[233]
NSC668394	small molecular inhibitor	inhibits Ezrin phosphorylation	melanoma	pre-clinical	[36]
NSC668394	small molecular inhibitor	inhibits Ezrin phosphorylation	hepatocellular carcinoma	pre-clinical	[234]
NSC305787	small molecular inhibitor	inhibits Ezrin phosphorylation	lung adenocarcinoma	in vitro	[235]
NSC668394+ Lapatinib	small molecular inhibitor	combination	breast cancer	pre-clinical	[131,236]
NSC668394+ DOX or DTX	small molecular inhibitor	combination	breast cancer	pre-clinical	[237]

## Data Availability

Not applicable.

## References

[1] Bretscher A. (1983). Purification of an 80,000-dalton protein that is a component of the isolated microvillus cytoskeleton, and its localization in nonmuscle cells. J. Cell Biol..

[2] Pakkanen R., Hedman K., Turunen O., Wahlström T., Vaheri A. (1987). Microvillus-specific Mr 75,000 plasma membrane protein of human choriocarcinoma cells. J. Histochem. Cytochem..

[3] Pakkanen R. (1988). Immunofluorescent and immunochemical evidence for the expression of cytovillin in the microvilli of a wide range of cultured human cells. J. Cell. Biochem..

[4] Urushidani T., Hanzel D.K., Forte J.G. (1989). Characterization of an 80-kDa phosphoprotein involved in parietal cell stimulation. Am. J. Physiol..

[5] Hunter T., Cooper J.A. (1981). Epidermal growth factor induces rapid tyrosine phosphorylation of proteins in A431 human tumor cells. Cell.

[6] Bretscher A. (1989). Rapid phosphorylation and reorganization of ezrin and spectrin accompany morphological changes induced in A-431 cells by epidermal growth factor. J. Cell Biol..

[7] Gould K.L., Bretscher A., Esch F.S., Hunter T. (1989). cDNA cloning and sequencing of the protein-tyrosine kinase substrate, ezrin, reveals homology to band 4.1. EMBO J..

[8] Gould K.L., Cooper J.A., Bretscher A., Hunter T. (1986). The protein-tyrosine kinase substrate, p81, is homologous to a chicken microvillar core protein. J. Cell Biol..

[9] Pakkanen R., Vaheri A. (1989). Cytovillin and other microvillar proteins of human choriocarcinoma cells. J. Cell. Biochem..

[10] Turunen O., Winqvist R., Pakkanen R., Grzeschik K.H., Wahlström T., Vaheri A. (1989). Cytovillin, a microvillar Mr 75,000 protein. cDNA sequence, prokaryotic expression, and chromosomal localization. J. Biol. Chem..

[11] Hanzel D., Reggio H., Bretscher A., Forte J.G., Mangeat P. (1991). The secretion-stimulated 80K phosphoprotein of parietal cells is ezrin, and has properties of a membrane cytoskeletal linker in the induced apical microvilli. EMBO J..

[12] Bretscher A., Reczek D., Berryman M. (1997). Ezrin: A protein requiring conformational activation to link microfilaments to the plasma membrane in the assembly of cell surface structures. J. Cell Sci..

[13] Srivastava J., Elliott B.E., Louvard D., Arpin M. (2005). Src-dependent ezrin phosphorylation in adhesion-mediated signaling. Mol. Biol. Cell.

[14] Gautreau A., Louvard D., Arpin M. (2002). ERM proteins and NF2 tumor suppressor: The Yin and Yang of cortical actin organization and cell growth signaling. Curr. Opin. Cell Biol..

[15] Mangeat P., Roy C., Martin M. (1999). ERM proteins in cell adhesion and membrane dynamics. Trends Cell Biol..

[16] Pujuguet P., Del Maestro L., Gautreau A., Louvard D., Arpin M. (2003). Ezrin regulates E-cadherin-dependent adherens junction assembly through Rac1 activation. Mol. Biol. Cell.

[17] Berryman M., Gary R., Bretscher A. (1995). Ezrin oligomers are major cytoskeletal components of placental microvilli: A proposal for their involvement in cortical morphogenesis. J. Cell Biol..

[18] Lamb R.F., Ozanne B.W., Roy C., McGarry L., Stipp C., Mangeat P., Jay D.G. (1997). Essential functions of ezrin in maintenance of cell shape and lamellipodial extension in normal and transformed fibroblasts. Curr. Biol..

[19] Mackay D.J., Esch F., Furthmayr H., Hall A. (1997). Rho- and rac-dependent assembly of focal adhesion complexes and actin filaments in permeabilized fibroblasts: An essential role for ezrin/radixin/moesin proteins. J. Cell Biol..

[20] Crepaldi T., Gautreau A., Comoglio P.M., Louvard D., Arpin M. (1997). Ezrin is an effector of hepatocyte growth factor-mediated migration and morphogenesis in epithelial cells. J. Cell Biol..

[21] Ng T., Parsons M., Hughes W.E., Monypenny J., Zicha D., Gautreau A., Arpin M., Gschmeissner S., Verveer P.J., Bastiaens P.I. (2001). Ezrin is a downstream effector of trafficking PKC-integrin complexes involved in the control of cell motility. EMBO J..

[22] Turunen O., Sainio M., Jääskeläinen J., Carpén O., Vaheri A. (1998). Structure-function relationships in the ezrin family and the effect of tumor-associated point mutations in neurofibromatosis 2 protein. Biochim. Biophys. Acta.

[23] Bretscher A., Edwards K., Fehon R.G. (2002). ERM proteins and merlin: Integrators at the cell cortex. Nat. Rev. Mol. Cell Biol..

[24] Algrain M., Turunen O., Vaheri A., Louvard D., Arpin M. (1993). Ezrin contains cytoskeleton and membrane binding domains accounting for its proposed role as a membrane-cytoskeletal linker. J. Cell Biol..

[25] Pearson M.A., Reczek D., Bretscher A., Karplus P.A. (2000). Structure of the ERM protein moesin reveals the FERM domain fold masked by an extended actin binding tail domain. Cell.

[26] Fehon R.G., McClatchey A.I., Bretscher A. (2010). Organizing the cell cortex: The role of ERM proteins. Nat. Rev. Mol. Cell Biol..

[27] Li J., Wei K., Yu H., Jin D., Wang G., Yu B. (2015). Prognostic Value of Ezrin in Various Cancers: A Systematic Review and Updated Meta-analysis. Sci. Rep..

[28] Steeg P.S. (2016). Targeting metastasis. Nat. Rev. Cancer.

[29] Chiang A.C., Massagué J. (2008). Molecular basis of metastasis. N. Engl. J. Med..

[30] Comen E., Norton L., Massagué J. (2011). Clinical implications of cancer self-seeding. Nat. Rev. Clin. Oncol..

[31] Pienta K.J., Robertson B.A., Coffey D.S., Taichman R.S. (2013). The cancer diaspora: Metastasis beyond the seed and soil hypothesis. Clin. Cancer Res..

[32] Luo W. (2023). Nasopharyngeal carcinoma ecology theory: Cancer as multidimensional spatiotemporal “unity of ecology and evolution” pathological ecosystem. Theranostics.

[33] Yin L.M., Schnoor M. (2022). Modulation of membrane-cytoskeleton interactions: Ezrin as key player. Trends Cell Biol..

[34] Barik G.K., Sahay O., Paul D., Santra M.K. (2022). Ezrin gone rogue in cancer progression and metastasis: An enticing therapeutic target. Biochim. Biophys. Acta Rev. Cancer.

[35] Orian-Rousseau V., Morrison H., Matzke A., Kastilan T., Pace G., Herrlich P., Ponta H. (2007). Hepatocyte growth factor-induced Ras activation requires ERM proteins linked to both CD44v6 and F-actin. Mol. Biol. Cell.

[36] Huang L., Qin Y., Zuo Q., Bhatnagar K., Xiong J., Merlino G., Yu Y. (2018). Ezrin mediates both HGF/Met autocrine and non-autocrine signaling-induced metastasis in melanoma. Int. J. Cancer.

[37] Saotome I., Curto M., McClatchey A.I. (2004). Ezrin is essential for epithelial organization and villus morphogenesis in the developing intestine. Dev. Cell.

[38] Tamura A., Kikuchi S., Hata M., Katsuno T., Matsui T., Hayashi H., Suzuki Y., Noda T., Tsukita S., Tsukita S. (2005). Achlorhydria by ezrin knockdown: Defects in the formation/expansion of apical canaliculi in gastric parietal cells. J. Cell Biol..

[39] Aoki K., Harada S., Kawaji K., Matsuzawa K., Uchida S., Ikenouchi J. (2021). STIM-Orai1 signaling regulates fluidity of cytoplasm during membrane blebbing. Nat. Commun..

[40] Song X., Wang W., Wang H., Yuan X., Yang F., Zhao L., Mullen M., Du S., Zohbi N., Muthusamy S. (2020). Acetylation of ezrin regulates membrane-cytoskeleton interaction underlying CCL18-elicited cell migration. J. Mol. Cell Biol..

[41] Welf E.S., Miles C.E., Huh J., Sapoznik E., Chi J., Driscoll M.K., Isogai T., Noh J., Weems A.D., Pohlkamp T. (2020). Actin-Membrane Release Initiates Cell Protrusions. Dev. Cell.

[42] Tsai F.C., Bertin A., Bousquet H., Manzi J., Senju Y., Tsai M.C., Picas L., Miserey-Lenkei S., Lappalainen P., Lemichez E. (2018). Ezrin enrichment on curved membranes requires a specific conformation or interaction with a curvature-sensitive partner. eLife.

[43] Berryman M., Franck Z., Bretscher A. (1993). Ezrin is concentrated in the apical microvilli of a wide variety of epithelial cells whereas moesin is found primarily in endothelial cells. J. Cell Sci..

[44] Bonilha V.L., Finnemann S.C., Rodriguez-Boulan E. (1999). Ezrin promotes morphogenesis of apical microvilli and basal infoldings in retinal pigment epithelium. J. Cell Biol..

[45] Casaletto J.B., Saotome I., Curto M., McClatchey A.I. (2011). Ezrin-mediated apical integrity is required for intestinal homeostasis. Proc. Natl. Acad. Sci. USA.

[46] Diz-Muñoz A., Krieg M., Bergert M., Ibarlucea-Benitez I., Muller D.J., Paluch E., Heisenberg C.P. (2010). Control of directed cell migration in vivo by membrane-to-cortex attachment. PLoS Biol..

[47] Liu Y., Belkina N.V., Park C., Nambiar R., Loughhead S.M., Patino-Lopez G., Ben-Aissa K., Hao J.J., Kruhlak M.J., Qi H. (2012). Constitutively active ezrin increases membrane tension, slows migration, and impedes endothelial transmigration of lymphocytes in vivo in mice. Blood.

[48] Rouven Brückner B., Pietuch A., Nehls S., Rother J., Janshoff A. (2015). Ezrin is a Major Regulator of Membrane Tension in Epithelial Cells. Sci. Rep..

[49] Braunger J.A., Brückner B.R., Nehls S., Pietuch A., Gerke V., Mey I., Janshoff A., Steinem C. (2014). Phosphatidylinositol 4,5-bisphosphate alters the number of attachment sites between ezrin and actin filaments: A colloidal probe study. J. Biol. Chem..

[50] Roberts R.E., Dewitt S., Hallett M.B. (2020). Membrane Tension and the Role of Ezrin During Phagocytosis. Adv. Exp. Med. Biol..

[51] Jia M., Yan X., Jiang X., Wu Y., Xu J., Meng Y., Yang Y., Shan X., Zhang X., Mao S. (2019). Ezrin, a Membrane Cytoskeleton Cross-Linker Protein, as a Marker of Epithelial Damage in Asthma. Am. J. Respir. Crit. Care Med..

[52] Lauffenburger D.A., Horwitz A.F. (1996). Cell migration: A physically integrated molecular process. Cell.

[53] Elumalai G.L., Dewitt S., Hallett M.B. (2011). Ezrin and talin relocates from the plasma membrane to cytosol during neutrophil extravasation. Eur. J. Clin. Investig..

[54] Elumalai G.L. (2012). Cytosolic Signalling and Behaviour of Oral Neutrophils “Search for Biochemical Memory”. Ph.D. Thesis.

[55] Peskin C.S., Odell G.M., Oster G.F. (1993). Cellular motions and thermal fluctuations: The Brownian ratchet. Biophys. J..

[56] Takeuchi K., Sato N., Kasahara H., Funayama N., Nagafuchi A., Yonemura S., Tsukita S., Tsukita S. (1994). Perturbation of cell adhesion and microvilli formation by antisense oligonucleotides to ERM family members. J. Cell Biol..

[57] Tsukita S., Oishi K., Sato N., Sagara J., Kawai A., Tsukita S. (1994). ERM family members as molecular linkers between the cell surface glycoprotein CD44 and actin-based cytoskeletons. J. Cell Biol..

[58] Yonemura S., Hirao M., Doi Y., Takahashi N., Kondo T., Tsukita S., Tsukita S. (1998). Ezrin/radixin/moesin (ERM) proteins bind to a positively charged amino acid cluster in the juxta-membrane cytoplasmic domain of CD44, CD43, and ICAM-2. J. Cell Biol..

[59] Heiska L., Alfthan K., Grönholm M., Vilja P., Vaheri A., Carpén O. (1998). Association of ezrin with intercellular adhesion molecule-1 and -2 (ICAM-1 and ICAM-2). Regulation by phosphatidylinositol 4, 5-bisphosphate. J. Biol. Chem..

[60] Kawaguchi K., Asano S. (2022). Pathophysiological Roles of Actin-Binding Scaffold Protein, Ezrin. Int. J. Mol. Sci..

[61] Freeman S.A., Vega A., Riedl M., Collins R.F., Ostrowski P.P., Woods E.C., Bertozzi C.R., Tammi M.I., Lidke D.S., Johnson P. (2018). Transmembrane Pickets Connect Cyto- and Pericellular Skeletons Forming Barriers to Receptor Engagement. Cell.

[62] Rey-Gallardo A., Tomlins H., Joachim J., Rahman I., Kitscha P., Frudd K., Parsons M., Ivetic A. (2018). Sequential binding of ezrin and moesin to L-selectin regulates monocyte protrusive behaviour during transendothelial migration. J. Cell Sci..

[63] Khan K., Long B., Deshpande G.M., Fox P.L. (2020). Bidirectional Tumor-Promoting Activities of Macrophage Ezrin. Int. J. Mol. Sci..

[64] Platet N., Prévostel C., Derocq D., Joubert D., Rochefort H., Garcia M. (1998). Breast cancer cell invasiveness: Correlation with protein kinase C activity and differential regulation by phorbol ester in estrogen receptor-positive and -negative cells. Int. J. Cancer.

[65] Sun X.G., Rotenberg S.A. (1999). Overexpression of protein kinase Calpha in MCF-10A human breast cells engenders dramatic alterations in morphology, proliferation, and motility. Cell Growth Differ..

[66] Zhang X., Flores L.R., Keeling M.C., Sliogeryte K., Gavara N. (2020). Ezrin Phosphorylation at T567 Modulates Cell Migration, Mechanical Properties, and Cytoskeletal Organization. Int. J. Mol. Sci..

[67] Kalluri R., Weinberg R.A. (2009). The basics of epithelial-mesenchymal transition. J. Clin. Investig..

[68] Fröse J., Chen M.B., Hebron K.E., Reinhardt F., Hajal C., Zijlstra A., Kamm R.D., Weinberg R.A. (2018). Epithelial-Mesenchymal Transition Induces Podocalyxin to Promote Extravasation via Ezrin Signaling. Cell Rep..

[69] Bunnell T.M., Burbach B.J., Shimizu Y., Ervasti J.M. (2011). β-Actin specifically controls cell growth, migration, and the G-actin pool. Mol. Biol. Cell.

[70] Gu Y., Tang S., Wang Z., Cai L., Lian H., Shen Y., Zhou Y. (2021). A pan-cancer analysis of the prognostic and immunological role of β-actin (ACTB) in human cancers. Bioengineered.

[71] Shuster C.B., Herman I.M. (1995). Indirect association of ezrin with F-actin: Isoform specificity and calcium sensitivity. J. Cell Biol..

[72] Gao Z.G., Inoue A., Jacobson K.A. (2018). On the G protein-coupling selectivity of the native A(2B) adenosine receptor. Biochem. Pharmacol..

[73] Sepúlveda C., Palomo I., Fuentes E. (2016). Role of adenosine A2b receptor overexpression in tumor progression. Life Sci..

[74] Sitaraman S.V., Wang L., Wong M., Bruewer M., Hobert M., Yun C.H., Merlin D., Madara J.L. (2002). The adenosine 2b receptor is recruited to the plasma membrane and associates with E3KARP and Ezrin upon agonist stimulation. J. Biol. Chem..

[75] Allen L.F., Lefkowitz R.J., Caron M.G., Cotecchia S. (1991). G-protein-coupled receptor genes as protooncogenes: Constitutively activating mutation of the alpha 1B-adrenergic receptor enhances mitogenesis and tumorigenicity. Proc. Natl. Acad. Sci. USA.

[76] Stanasila L., Abuin L., Diviani D., Cotecchia S. (2006). Ezrin directly interacts with the alpha1b-adrenergic receptor and plays a role in receptor recycling. J. Biol. Chem..

[77] D’Souza-Schorey C., Chavrier P. (2006). ARF proteins: Roles in membrane traffic and beyond. Nat. Rev. Mol. Cell Biol..

[78] Macia E., Luton F., Partisani M., Cherfils J., Chardin P., Franco M. (2004). The GDP-bound form of Arf6 is located at the plasma membrane. J. Cell Sci..

[79] Fang Z., Miao Y., Ding X., Deng H., Liu S., Wang F., Zhou R., Watson C., Fu C., Hu Q. (2006). Proteomic identification and functional characterization of a novel ARF6 GTPase-activating protein, ACAP4. Mol. Cell Proteom..

[80] Ding X., Deng H., Wang D., Zhou J., Huang Y., Zhao X., Yu X., Wang M., Wang F., Ward T. (2010). Phospho-regulated ACAP4-Ezrin interaction is essential for histamine-stimulated parietal cell secretion. J. Biol. Chem..

[81] Takai Y., Sasaki T., Tanaka K., Nakanishi H. (1995). Rho as a regulator of the cytoskeleton. Trends Biochem. Sci..

[82] Takahashi K., Sasaki T., Mammoto A., Takaishi K., Kameyama T., Tsukita S., Takai Y. (1997). Direct interaction of the Rho GDP dissociation inhibitor with ezrin/radixin/moesin initiates the activation of the Rho small G protein. J. Biol. Chem..

[83] Luo Y., Zheng C., Zhang J., Lu D., Zhuang J., Xing S., Feng J., Yang D., Yan X. (2012). Recognition of CD146 as an ERM-binding protein offers novel mechanisms for melanoma cell migration. Oncogene.

[84] Ota T., Maeda M., Suto S., Tatsuka M. (2004). LyGDI functions in cancer metastasis by anchoring Rho proteins to the cell membrane. Mol. Carcinog..

[85] Sainio M., Zhao F., Heiska L., Turunen O., den Bakker M., Zwarthoff E., Lutchman M., Rouleau G.A., Jääskeläinen J., Vaheri A. (1997). Neurofibromatosis 2 tumor suppressor protein colocalizes with ezrin and CD44 and associates with actin-containing cytoskeleton. J. Cell Sci..

[86] Ren M., Zhao L., Ma Z., An H., Marrink S.J., Sun F. (2023). Molecular basis of PIP2-dependent conformational switching of phosphorylated CD44 in binding FERM. Biophys. J..

[87] Herrlich P., Morrison H., Sleeman J., Orian-Rousseau V., König H., Weg-Remers S., Ponta H. (2000). CD44 acts both as a growth- and invasiveness-promoting molecule and as a tumor-suppressing cofactor. Ann. N. Y. Acad. Sci..

[88] Zohar R., Suzuki N., Suzuki K., Arora P., Glogauer M., McCulloch C.A., Sodek J. (2000). Intracellular osteopontin is an integral component of the CD44-ERM complex involved in cell migration. J. Cell Physiol..

[89] Martin T.A., Harrison G., Mansel R.E., Jiang W.G. (2003). The role of the CD44/ezrin complex in cancer metastasis. Crit. Rev. Oncol. Hematol..

[90] Takeichi M. (1993). Cadherins in cancer: Implications for invasion and metastasis. Curr. Opin. Cell Biol..

[91] Hiscox S., Jiang W.G. (1999). Ezrin regulates cell-cell and cell-matrix adhesion, a possible role with E-cadherin/beta-catenin. J. Cell Sci..

[92] Yao W., Feng D., Bian W., Yang L., Li Y., Yang Z., Xiong Y., Zheng J., Zhai R., He J. (2012). EBP50 inhibits EGF-induced breast cancer cell proliferation by blocking EGFR phosphorylation. Amino Acids.

[93] Yu Z., Sun M., Jin F., Xiao Q., He M., Wu H., Ren J., Zhao L., Zhao H., Yao W. (2015). Combined expression of ezrin and E-cadherin is associated with lymph node metastasis and poor prognosis in breast cancer. Oncol. Rep..

[94] Guedj N., Vaquero J., Clapéron A., Mergey M., Chrétien Y., Paradis V., Fouassier L. (2016). Loss of ezrin in human intrahepatic cholangiocarcinoma is associated with ectopic expression of E-cadherin. Histopathology.

[95] Berryman M., Bretscher A. (2000). Identification of a novel member of the chloride intracellular channel gene family (CLIC5) that associates with the actin cytoskeleton of placental microvilli. Mol. Biol. Cell.

[96] Wegner B., Al-Momany A., Kulak S.C., Kozlowski K., Obeidat M., Jahroudi N., Paes J., Berryman M., Ballermann B.J. (2010). CLIC5A, a component of the ezrin-podocalyxin complex in glomeruli, is a determinant of podocyte integrity. Am. J. Physiol. Renal Physiol..

[97] Pierchala B.A., Muñoz M.R., Tsui C.C. (2010). Proteomic analysis of the slit diaphragm complex: CLIC5 is a protein critical for podocyte morphology and function. Kidney Int..

[98] Al-Momany A., Li L., Alexander R.T., Ballermann B.J. (2014). Clustered PI(4,5)P_2_ accumulation and ezrin phosphorylation in response to CLIC5A. J. Cell Sci..

[99] Flores-Téllez T.N., Lopez T.V., Vásquez Garzón V.R., Villa-Treviño S. (2015). Co-Expression of Ezrin-CLIC5-Podocalyxin Is Associated with Migration and Invasiveness in Hepatocellular Carcinoma. PLoS ONE.

[100] Gavert N., Ben-Ze’ev A. (2007). beta-Catenin signaling in biological control and cancer. J. Cell Biochem..

[101] Gavert N., Ben-Shmuel A., Lemmon V., Brabletz T., Ben-Ze’ev A. (2010). Nuclear factor-kappaB signaling and ezrin are essential for L1-mediated metastasis of colon cancer cells. J. Cell Sci..

[102] Khazaie K., Schirrmacher V., Lichtner R.B. (1993). EGF receptor in neoplasia and metastasis. Cancer Metastasis Rev..

[103] Chiasson-MacKenzie C., Morris Z.S., Baca Q., Morris B., Coker J.K., Mirchev R., Jensen A.E., Carey T., Stott S.L., Golan D.E. (2015). NF2/Merlin mediates contact-dependent inhibition of EGFR mobility and internalization via cortical actomyosin. J. Cell Biol..

[104] Saygideğer-Kont Y., Minas T.Z., Jones H., Hour S., Çelik H., Temel I., Han J., Atabey N., Erkizan H.V., Toretsky J.A. (2016). Ezrin Enhances EGFR Signaling and Modulates Erlotinib Sensitivity in Non-Small Cell Lung Cancer Cells. Neoplasia.

[105] Kischkel F.C., Hellbardt S., Behrmann I., Germer M., Pawlita M., Krammer P.H., Peter M.E. (1995). Cytotoxicity-dependent APO-1 (Fas/CD95)-associated proteins form a death-inducing signaling complex (DISC) with the receptor. EMBO J..

[106] Parlato S., Giammarioli A.M., Logozzi M., Lozupone F., Matarrese P., Luciani F., Falchi M., Malorni W., Fais S. (2000). CD95 (APO-1/Fas) linkage to the actin cytoskeleton through ezrin in human T lymphocytes: A novel regulatory mechanism of the CD95 apoptotic pathway. EMBO J..

[107] Kuo W.C., Yang K.T., Hsieh S.L., Lai M.Z. (2010). Ezrin is a negative regulator of death receptor-induced apoptosis. Oncogene.

[108] Gahmberg C.G., Tolvanen M., Kotovuori P. (1997). Leukocyte adhesion—Structure and function of human leukocyte beta2-integrins and their cellular ligands. Eur. J. Biochem..

[109] Helander T.S., Carpén O., Turunen O., Kovanen P.E., Vaheri A., Timonen T. (1996). ICAM-2 redistributed by ezrin as a target for killer cells. Nature.

[110] Serrador J.M., Alonso-Lebrero J.L., del Pozo M.A., Furthmayr H., Schwartz-Albiez R., Calvo J., Lozano F., Sánchez-Madrid F. (1997). Moesin interacts with the cytoplasmic region of intercellular adhesion molecule-3 and is redistributed to the uropod of T lymphocytes during cell polarization. J. Cell Biol..

[111] Kong J., Yao C., Dong S., Wu S., Xu Y., Li K., Ji L., Shen Q., Zhang Q., Zhan R. (2021). ICAM-1 Activates Platelets and Promotes Endothelial Permeability through VE-Cadherin after Insufficient Radiofrequency Ablation. Adv. Sci..

[112] White C.D., Erdemir H.H., Sacks D.B. (2012). IQGAP1 and its binding proteins control diverse biological functions. Cell Signal.

[113] Peng X., Wang T., Gao H., Yue X., Bian W., Mei J., Zhang Y. (2021). The interplay between IQGAP1 and small GTPases in cancer metastasis. Biomed. Pharmacother..

[114] Nammalwar R.C., Heil A., Gerke V. (2015). Ezrin interacts with the scaffold protein IQGAP1 and affects its cortical localization. Biochim. Biophys. Acta.

[115] Liu J., Guidry J.J., Worthylake D.K. (2014). Conserved sequence repeats of IQGAP1 mediate binding to Ezrin. J. Proteome Res..

[116] Weinspach D., Seubert B., Schaten S., Honert K., Sebens S., Altevogt P., Krüger A. (2014). Role of L1 cell adhesion molecule (L1CAM) in the metastatic cascade: Promotion of dissemination, colonization, and metastatic growth. Clin. Exp. Metastasis.

[117] Guo J.C., Xie Y.M., Ran L.Q., Cao H.H., Sun C., Wu J.Y., Wu Z.Y., Liao L.D., Zhao W.J., Fang W.K. (2017). L1CAM drives oncogenicity in esophageal squamous cell carcinoma by stimulation of ezrin transcription. J. Mol. Med..

[118] Kiefel H., Bondong S., Pfeifer M., Schirmer U., Erbe-Hoffmann N., Schäfer H., Sebens S., Altevogt P. (2012). EMT-associated up-regulation of L1CAM provides insights into L1CAM-mediated integrin signalling and NF-κB activation. Carcinogenesis.

[119] Maccio U., Mihic A., Lenggenhager D., Kolm I., Mittmann C., Heikenwälder M., Lorentzen A., Mihic-Probst D. (2022). Hypoxia and Ezrin Expression in Primary Melanoma Have High Prognostic Relevance. Int. J. Mol. Sci..

[120] Shcherbina A., Bretscher A., Kenney D.M., Remold-O’Donnell E. (1999). Moesin, the major ERM protein of lymphocytes and platelets, differs from ezrin in its insensitivity to calpain. FEBS Lett..

[121] Parameswaran N., Gupta N. (2013). Re-defining ERM function in lymphocyte activation and migration. Immunol. Rev..

[122] Xu H.M., Gutmann D.H. (1998). Merlin differentially associates with the microtubule and actin cytoskeleton. J. Neurosci. Res..

[123] Stamenkovic I., Yu Q. (2010). Merlin, a “magic” linker between extracellular cues and intracellular signaling pathways that regulate cell motility, proliferation, and survival. Curr. Protein Pept. Sci..

[124] Ye K. (2007). Phosphorylation of merlin regulates its stability and tumor suppressive activity. Cell Adh. Migr..

[125] Muranen T., Grönholm M., Lampin A., Lallemand D., Zhao F., Giovannini M., Carpén O. (2007). The tumor suppressor merlin interacts with microtubules and modulates Schwann cell microtubule cytoskeleton. Hum. Mol. Genet..

[126] Luo Z.L., Cheng S.Q., Shi J., Zhang H.L., Zhang C.Z., Chen H.Y., Qiu B.J., Tang L., Hu C.L., Wang H.Y. (2015). A splicing variant of Merlin promotes metastasis in hepatocellular carcinoma. Nat. Commun..

[127] Zhou J., Feng Y., Tao K., Su Z., Yu X., Zheng J., Zhang L., Yang D. (2014). The expression and phosphorylation of ezrin and merlin in human pancreatic cancer. Int. J. Oncol..

[128] Morales F.C., Molina J.R., Hayashi Y., Georgescu M.M. (2010). Overexpression of ezrin inactivates NF2 tumor suppressor in glioblastoma. Neuro Oncol..

[129] Otey C.A., Rachlin A., Moza M., Arneman D., Carpen O. (2005). The palladin/myotilin/myopalladin family of actin-associated scaffolds. Int. Rev. Cytol..

[130] Mykkänen O.M., Grönholm M., Rönty M., Lalowski M., Salmikangas P., Suila H., Carpén O. (2001). Characterization of human palladin, a microfilament-associated protein. Mol. Biol. Cell.

[131] Jeong J., Choi J., Kim W., Dann P., Takyar F., Gefter J.V., Friedman P.A., Wysolmerski J.J. (2019). Inhibition of ezrin causes PKCα-mediated internalization of erbb2/HER2 tyrosine kinase in breast cancer cells. J. Biol. Chem..

[132] Schwock J., Dhani N., Hedley D.W. (2010). Targeting focal adhesion kinase signaling in tumor growth and metastasis. Expert Opin. Ther. Targets.

[133] Golubovskaya V.M. (2014). Targeting FAK in human cancer: From finding to first clinical trials. Front. Biosci..

[134] Paul R., Luo M., Mo X., Lu J., Yeo S.K., Guan J.L. (2020). FAK activates AKT-mTOR signaling to promote the growth and progression of MMTV-Wnt1-driven basal-like mammary tumors. Breast Cancer Res..

[135] Poullet P., Gautreau A., Kadaré G., Girault J.A., Louvard D., Arpin M. (2001). Ezrin interacts with focal adhesion kinase and induces its activation independently of cell-matrix adhesion. J. Biol. Chem..

[136] Xu J., Zhang W. (2021). EZR promotes pancreatic cancer proliferation and metastasis by activating FAK/AKT signaling pathway. Cancer Cell Int..

[137] Jiang Q.H., Wang A.X., Chen Y. (2014). Radixin enhances colon cancer cell invasion by increasing MMP-7 production via Rac1-ERK pathway. Sci. World J..

[138] Bukong T.N., Kodys K., Szabo G. (2013). Human ezrin-moesin-radixin proteins modulate hepatitis C virus infection. Hepatology.

[139] Qin J.J., Wang J.M., Du J., Zeng C., Han W., Li Z.D., Xie J., Li G.L. (2014). Radixin knockdown by RNA interference suppresses human glioblastoma cell growth in vitro and in vivo. Asian Pac. J. Cancer Prev..

[140] Zhu Y.W., Yan J.K., Li J.J., Ou Y.M., Yang Q. (2016). Knockdown of Radixin Suppresses Gastric Cancer Metastasis In Vitro by Up-Regulation of E-Cadherin via NF-κB/Snail Pathway. Cell Physiol. Biochem..

[141] Yuan J., Xiao C., Lu H., Yu H., Hong H., Guo C., Wu Z. (2020). miR-200b regulates breast cancer cell proliferation and invasion by targeting radixin. Exp. Ther. Med..

[142] Schmieder S., Nagai M., Orlando R.A., Takeda T., Farquhar M.G. (2004). Podocalyxin activates RhoA and induces actin reorganization through NHERF1 and Ezrin in MDCK cells. J. Am. Soc. Nephrol..

[143] Ma L., Liu Y.P., Zhang X.H., Geng C.Z., Li Z.H. (2013). Relationship of RhoA signaling activity with ezrin expression and its significance in the prognosis for breast cancer patients. Chin. Med. J..

[144] Ma L., Liu Y.P., Zhang X.H., Xing L.X., Wang J.L., Geng C.Z. (2009). Effect of RhoA signaling transduction on expression of Ezrin in breast cancer cell lines. Ai Zheng.

[145] Chiappetta C., Leopizzi M., Censi F., Puggioni C., Petrozza V., Rocca C.D., Di Cristofano C. (2014). Correlation of the Rac1/RhoA pathway with ezrin expression in osteosarcoma. Appl. Immunohistochem. Mol. Morphol..

[146] Li M.J., Xiong D., Huang H., Wen Z.Y. (2021). Ezrin Promotes the Proliferation, Migration, and Invasion of Ovarian Cancer Cells. Biomed. Environ. Sci..

[147] Rath N., Olson M.F. (2012). Rho-associated kinases in tumorigenesis: Re-considering ROCK inhibition for cancer therapy. EMBO Rep..

[148] Tsuda M., Makino Y., Iwahara T., Nishihara H., Sawa H., Nagashima K., Hanafusa H., Tanaka S. (2004). Crk associates with ERM proteins and promotes cell motility toward hyaluronic acid. J. Biol. Chem..

[149] Ding N., Li P., Li H., Lei Y., Zhang Z. (2022). The ROCK-ezrin signaling pathway mediates LPS-induced cytokine production in pulmonary alveolar epithelial cells. Cell Commun. Signal.

[150] Hébert M., Potin S., Sebbagh M., Bertoglio J., Bréard J., Hamelin J. (2008). Rho-ROCK-dependent ezrin-radixin-moesin phosphorylation regulates Fas-mediated apoptosis in Jurkat cells. J. Immunol..

[151] Koltzscher M., Neumann C., König S., Gerke V. (2003). Ca^2+^-dependent binding and activation of dormant ezrin by dimeric S100P. Mol. Biol Cell.

[152] Austermann J., Nazmi A.R., Müller-Tidow C., Gerke V. (2008). Characterization of the Ca2+ -regulated ezrin-S100P interaction and its role in tumor cell migration. J. Biol. Chem..

[153] Sullivan A., Uff C.R., Isacke C.M., Thorne R.F. (2003). PACE-1, a novel protein that interacts with the C-terminal domain of ezrin. Exp. Cell Res..

[154] Granés F., Berndt C., Roy C., Mangeat P., Reina M., Vilaró S. (2003). Identification of a novel Ezrin-binding site in syndecan-2 cytoplasmic domain. FEBS Lett..

[155] Mytilinaiou M., Nikitovic D., Berdiaki A., Papoutsidakis A., Papachristou D.J., Tsatsakis A., Tzanakakis G.N. (2017). IGF-I regulates HT1080 fibrosarcoma cell migration through a syndecan-2/Erk/ezrin signaling axis. Exp. Cell Res..

[156] Ivetic A., Florey O., Deka J., Haskard D.O., Ager A., Ridley A.J. (2004). Mutagenesis of the ezrin-radixin-moesin binding domain of L-selectin tail affects shedding, microvillar positioning, and leukocyte tethering. J. Biol. Chem..

[157] Martinelli S., Chen E.J., Clarke F., Lyck R., Affentranger S., Burkhardt J.K., Niggli V. (2013). Ezrin/Radixin/Moesin proteins and flotillins cooperate to promote uropod formation in T cells. Front. Immunol..

[158] Borsig L. (2018). Selectins in cancer immunity. Glycobiology.

[159] Cardone R.A., Casavola V., Reshkin S.J. (2005). The role of disturbed pH dynamics and the Na+/H+ exchanger in metastasis. Nat. Rev. Cancer.

[160] Chiang Y., Chou C.Y., Hsu K.F., Huang Y.F., Shen M.R. (2008). EGF upregulates Na+/H+ exchanger NHE1 by post-translational regulation that is important for cervical cancer cell invasiveness. J. Cell Physiol..

[161] Lauritzen G., Stock C.M., Lemaire J., Lund S.F., Jensen M.F., Damsgaard B., Petersen K.S., Wiwel M., Rønnov-Jessen L., Schwab A. (2012). The Na+/H+ exchanger NHE1, but not the Na+, HCO3(-) cotransporter NBCn1, regulates motility of MCF7 breast cancer cells expressing constitutively active ErbB2. Cancer Lett..

[162] Stock C., Ludwig F.T., Schwab A. (2012). Is the multifunctional Na(+)/H(+) exchanger isoform 1 a potential therapeutic target in cancer?. Curr. Med. Chem..

[163] Frontzek F., Nitzlaff S., Horstmann M., Schwab A., Stock C. (2014). Functional interdependence of NHE1 and merlin in human melanoma cells. Biochem. Cell Biol..

[164] Denker S.P., Barber D.L. (2002). Cell migration requires both ion translocation and cytoskeletal anchoring by the Na-H exchanger NHE1. J. Cell Biol..

[165] Denker S.P., Huang D.C., Orlowski J., Furthmayr H., Barber D.L. (2000). Direct binding of the Na-H exchanger NHE1 to ERM proteins regulates the cortical cytoskeleton and cell shape independently of H(+) translocation. Mol. Cell.

[166] Wu K.L., Khan S., Lakhe-Reddy S., Jarad G., Mukherjee A., Obejero-Paz C.A., Konieczkowski M., Sedor J.R., Schelling J.R. (2004). The NHE1 Na+/H+ exchanger recruits ezrin/radixin/moesin proteins to regulate Akt-dependent cell survival. J. Biol. Chem..

[167] Malo M.E., Fliegel L. (2006). Physiological role and regulation of the Na+/H+ exchanger. Can. J. Physiol. Pharmacol..

[168] Bera K., Kiepas A., Godet I., Li Y., Mehta P., Ifemembi B., Paul C.D., Sen A., Serra S.A., Stoletov K. (2022). Extracellular fluid viscosity enhances cell migration and cancer dissemination. Nature.

[169] Vaquero J., Nguyen Ho-Bouldoires T.H., Clapéron A., Fouassier L. (2017). Role of the PDZ-scaffold protein NHERF1/EBP50 in cancer biology: From signaling regulation to clinical relevance. Oncogene.

[170] Vondriska T.M., Pass J.M., Ping P. (2004). Scaffold proteins and assembly of multiprotein signaling complexes. J. Mol. Cell Cardiol..

[171] Dard N., Peter M. (2006). Scaffold proteins in MAP kinase signaling: More than simple passive activating platforms. Bioessays.

[172] Clapéron A., Therrien M. (2007). KSR and CNK: Two scaffolds regulating RAS-mediated RAF activation. Oncogene.

[173] Hsu Y.H., Lin W.L., Hou Y.T., Pu Y.S., Shun C.T., Chen C.L., Wu Y.Y., Chen J.Y., Chen T.H., Jou T.S. (2010). Podocalyxin EBP50 ezrin molecular complex enhances the metastatic potential of renal cell carcinoma through recruiting Rac1 guanine nucleotide exchange factor ARHGEF7. Am. J. Pathol..

[174] Yun C.H., Lamprecht G., Forster D.V., Sidor A. (1998). NHE3 kinase A regulatory protein E3KARP binds the epithelial brush border Na+/H+ exchanger NHE3 and the cytoskeletal protein ezrin. J. Biol. Chem..

[175] Lamprecht G., Weinman E.J., Yun C.H. (1998). The role of NHERF and E3KARP in the cAMP-mediated inhibition of NHE3. J. Biol. Chem..

[176] Zizak M., Lamprecht G., Steplock D., Tariq N., Shenolikar S., Donowitz M., Yun C.H., Weinman E.J. (1999). cAMP-induced phosphorylation and inhibition of Na(+)/H(+) exchanger 3 (NHE3) are dependent on the presence but not the phosphorylation of NHE regulatory factor. J. Biol. Chem..

[177] Ingraffea J., Reczek D., Bretscher A. (2002). Distinct cell type-specific expression of scaffolding proteins EBP50 and E3KARP: EBP50 is generally expressed with ezrin in specific epithelia, whereas E3KARP is not. Eur. J. Cell Biol..

[178] Boratkó A., Csortos C. (2013). NHERF2 is crucial in ERM phosphorylation in pulmonary endothelial cells. Cell Commun. Signal.

[179] Orlando R.A., Takeda T., Zak B., Schmieder S., Benoit V.M., McQuistan T., Furthmayr H., Farquhar M.G. (2001). The glomerular epithelial cell anti-adhesin podocalyxin associates with the actin cytoskeleton through interactions with ezrin. J. Am. Soc. Nephrol..

[180] Hatano R., Takeda A., Abe Y., Kawaguchi K., Kazama I., Matsubara M., Asano S. (2018). Loss of ezrin expression reduced the susceptibility to the glomerular injury in mice. Sci. Rep..

[181] Fukusumi Y., Yasuda H., Zhang Y., Kawachi H. (2021). Nephrin-Ephrin-B1-Na(+)/H(+) Exchanger Regulatory Factor 2-Ezrin-Actin Axis Is Critical in Podocyte Injury. Am. J. Pathol..

[182] Ghai R., Tello-Lafoz M., Norwood S.J., Yang Z., Clairfeuille T., Teasdale R.D., Mérida I., Collins B.M. (2015). Phosphoinositide binding by the SNX27 FERM domain regulates its localization at the immune synapse of activated T-cells. J. Cell Sci..

[183] Tello-Lafoz M., Ghai R., Collins B., Mérida I. (2014). A role for novel lipid interactions in the dynamic recruitment of SNX27 to the T-cell immune synapse. Bioarchitecture.

[184] Ghai R., Mobli M., Norwood S.J., Bugarcic A., Teasdale R.D., King G.F., Collins B.M. (2011). Phox homology band 4.1/ezrin/radixin/moesin-like proteins function as molecular scaffolds that interact with cargo receptors and Ras GTPases. Proc. Natl. Acad. Sci. USA.

[185] Chandra M., Kendall A.K., Jackson L.P. (2021). Toward Understanding the Molecular Role of SNX27/Retromer in Human Health and Disease. Front. Cell Dev. Biol..

[186] Zhang J., Li K., Zhang Y., Lu R., Wu S., Tang J., Xia Y., Sun J. (2019). Deletion of sorting nexin 27 suppresses proliferation in highly aggressive breast cancer MDA-MB-231 cells in vitro and in vivo. BMC Cancer.

[187] Tong J., Allenspach E.J., Takahashi S.M., Mody P.D., Park C., Burkhardt J.K., Sperling A.I. (2004). CD43 regulation of T cell activation is not through steric inhibition of T cell-APC interactions but through an intracellular mechanism. J. Exp. Med..

[188] Serrador J.M., Nieto M., Alonso-Lebrero J.L., del Pozo M.A., Calvo J., Furthmayr H., Schwartz-Albiez R., Lozano F., González-Amaro R., Sánchez-Mateos P. (1998). CD43 interacts with moesin and ezrin and regulates its redistribution to the uropods of T lymphocytes at the cell-cell contacts. Blood.

[189] Cannon J.L., Mody P.D., Blaine K.M., Chen E.J., Nelson A.D., Sayles L.J., Moore T.V., Clay B.S., Dulin N.O., Shilling R.A. (2011). CD43 interaction with ezrin-radixin-moesin (ERM) proteins regulates T-cell trafficking and CD43 phosphorylation. Mol. Biol. Cell.

[190] Yonemura S., Tsukita S., Tsukita S. (1999). Direct involvement of ezrin/radixin/moesin (ERM)-binding membrane proteins in the organization of microvilli in collaboration with activated ERM proteins. J. Cell Biol..

[191] Lamb R.F., Roy C., Diefenbach T.J., Vinters H.V., Johnson M.W., Jay D.G., Hall A. (2000). The TSC1 tumour suppressor hamartin regulates cell adhesion through ERM proteins and the GTPase Rho. Nat. Cell Biol..

[192] Ognibene M., Vanni C., Segalerba D., Mancini P., Merello E., Torrisi M.R., Bosco M.C., Varesio L., Eva A. (2011). The tumor suppressor hamartin enhances Dbl protein transforming activity through interaction with ezrin. J. Biol. Chem..

[193] Barreiro O., Yanez-Mo M., Serrador J.M., Montoya M.C., Vicente-Manzanares M., Tejedor R., Furthmayr H., Sanchez-Madrid F. (2002). Dynamic interaction of VCAM-1 and ICAM-1 with moesin and ezrin in a novel endothelial docking structure for adherent leukocytes. J. Cell Biol..

[194] Chen Q., Zhang X.H., Massagué J. (2011). Macrophage binding to receptor VCAM-1 transmits survival signals in breast cancer cells that invade the lungs. Cancer Cell.

[195] Bretscher A. (1999). Regulation of cortical structure by the ezrin-radixin-moesin protein family. Curr. Opin. Cell Biol..

[196] Fais S. (2004). A role for ezrin in a neglected metastatic tumor function. Trends Mol. Med..

[197] Marin-Padilla M. (1977). Erythrophagocytosis by epithelial cells of a breast carcinoma. Cancer.

[198] DeSimone P.A., East R., Powell R.D. (1980). Phagocytic tumor cell activity in oat cell carcinoma of the lung. Hum. Pathol..

[199] Defacque H., Egeberg M., Habermann A., Diakonova M., Roy C., Mangeat P., Voelter W., Marriott G., Pfannstiel J., Faulstich H. (2000). Involvement of ezrin/moesin in de novo actin assembly on phagosomal membranes. EMBO J..

[200] Lugini L., Lozupone F., Matarrese P., Funaro C., Luciani F., Malorni W., Rivoltini L., Castelli C., Tinari A., Piris A. (2003). Potent phagocytic activity discriminates metastatic and primary human malignant melanomas: A key role of ezrin. Lab. Investig..

[201] Lugini L., Matarrese P., Tinari A., Lozupone F., Federici C., Iessi E., Gentile M., Luciani F., Parmiani G., Rivoltini L. (2006). Cannibalism of live lymphocytes by human metastatic but not primary melanoma cells. Cancer Res..

[202] Chang Y.T., Peng H.Y., Hu C.M., Huang S.C., Tien S.C., Jeng Y.M. (2020). Pancreatic cancer-derived small extracellular vesical Ezrin regulates macrophage polarization and promotes metastasis. Am. J. Cancer Res..

[203] Chávez-Galán L., Olleros M.L., Vesin D., Garcia I. (2015). Much More than M1 and M2 Macrophages, There are also CD169(+) and TCR(+) Macrophages. Front. Immunol..

[204] Charrin S., Alcover A. (2006). Role of ERM (ezrin-radixin-moesin) proteins in T lymphocyte polarization, immune synapse formation and in T cell receptor-mediated signaling. Front. Biosci..

[205] García-Ortiz A., Serrador J.M. (2020). ERM Proteins at the Crossroad of Leukocyte Polarization, Migration and Intercellular Adhesion. Int. J. Mol. Sci..

[206] Kakurina G.V., Stakheeva M.N., Bakhronov I.A., Sereda E.E., Cheremisina O.V., Choynzonov E.L., Kondakova I.V. (2021). Circulating Actin-Binding Proteins in Laryngeal Cancer: Its Relationship with Circulating Tumor Cells and Cells of the Immune System. Acta Nat..

[207] Gatalica Z., Snyder C., Maney T., Ghazalpour A., Holterman D.A., Xiao N., Overberg P., Rose I., Basu G.D., Vranic S. (2014). Programmed cell death 1 (PD-1) and its ligand (PD-L1) in common cancers and their correlation with molecular cancer type. Cancer Epidemiol. Biomark. Prev..

[208] Tu X., Qin B., Zhang Y., Zhang C., Kahila M., Nowsheen S., Yin P., Yuan J., Pei H., Li H. (2019). PD-L1 (B7-H1) Competes with the RNA Exosome to Regulate the DNA Damage Response and Can Be Targeted to Sensitize to Radiation or Chemotherapy. Mol. Cell.

[209] Kobori T., Tanaka C., Tameishi M., Urashima Y., Ito T., Obata T. (2021). Role of Ezrin/Radixin/Moesin in the Surface Localization of Programmed Cell Death Ligand-1 in Human Colon Adenocarcinoma LS180 Cells. Pharmaceuticals.

[210] Tanaka C., Kobori T., Tameishi M., Urashima Y., Ito T., Obata T. (2021). Ezrin Modulates the Cell Surface Expression of Programmed Cell Death Ligand-1 in Human Cervical Adenocarcinoma Cells. Molecules.

[211] Tanaka C., Kobori T., Okada R., Doukuni R., Tameishi M., Urashima Y., Ito T., Takagaki N., Obata T. (2022). Ezrin Regulates the Cell Surface Localization of PD-L1 in HEC-151 Cells. J. Clin. Med..

[212] Yu Y. (2023). The Function of NK Cells in Tumor Metastasis and NK Cell-Based Immunotherapy. Cancers.

[213] Wang S., Guo Z., Xia P., Liu T., Wang J., Li S., Sun L., Lu J., Wen Q., Zhou M. (2009). Internalization of NK cells into tumor cells requires ezrin and leads to programmed cell-in-cell death. Cell Res..

[214] Satooka H., Matsui M., Ichioka S., Nakamura Y., Hirata T. (2022). The ERM protein moesin regulates natural killer cell homeostasis in vivo. Cell Immunol..

[215] Yu Y., Khan J., Khanna C., Helman L., Meltzer P.S., Merlino G. (2004). Expression profiling identifies the cytoskeletal organizer ezrin and the developmental homeoprotein Six-1 as key metastatic regulators. Nat. Med..

[216] Kong J., Di C., Piao J., Sun J., Han L., Chen L., Yan G., Lin Z. (2016). Ezrin contributes to cervical cancer progression through induction of epithelial-mesenchymal transition. Oncotarget.

[217] Li Y., Lin Z., Chen B., Chen S., Jiang Z., Zhou T., Hou Z., Wang Y. (2017). Ezrin/NF-kB activation regulates epithelial-mesenchymal transition induced by EGF and promotes metastasis of colorectal cancer. Biomed. Pharmacother..

[218] Zhong G.X., Feng S.D., Shen R., Wu Z.Y., Chen F., Zhu X. (2017). The clinical significance of the Ezrin gene and circulating tumor cells in osteosarcoma. OncoTargets Ther..

[219] Hoskin V., Szeto A., Ghaffari A., Greer P.A., Côté G.P., Elliott B.E. (2015). Ezrin regulates focal adhesion and invadopodia dynamics by altering calpain activity to promote breast cancer cell invasion. Mol. Biol. Cell.

[220] Bartova M., Hlavaty J., Tan Y., Singer C., Pohlodek K., Luha J., Walter I. (2017). Expression of ezrin and moesin in primary breast carcinoma and matched lymph node metastases. Clin. Exp. Metastasis.

[221] Chen M., Pan Y., Liu H., Ning F., Lu Q., Duan Y., Gan X., Lu S., Hou H., Zhang M. (2022). Ezrin accelerates breast cancer liver metastasis through promoting furin-like convertase-mediated cleavage of Notch1. Cell Oncol..

[222] Zhan X.H., Jiao J.W., Zhang H.F., Xu X.E., He J.Z., Li R.L., Zou H.Y., Wu Z.Y., Wang S.H., Wu J.Y. (2019). LOXL2 Upregulates Phosphorylation of Ezrin to Promote Cytoskeletal Reorganization and Tumor Cell Invasion. Cancer Res..

[223] Chen Z., Wang J., Lu Y., Lai C., Qu L., Zhuo Y. (2022). Ezrin expression in circulating tumor cells is a predictor of prostate cancer metastasis. Bioengineered.

[224] Tang Y., Sun X., Yu S., Bie X., Wang J., Ren L. (2019). Inhibition of Ezrin suppresses cell migration and invasion in human nasopharyngeal carcinoma. Oncol. Lett..

[225] Yu Y., Davicioni E., Triche T.J., Merlino G. (2006). The homeoprotein six1 transcriptionally activates multiple protumorigenic genes but requires ezrin to promote metastasis. Cancer Res..

[226] Gautreau A., Louvard D., Arpin M. (2000). Morphogenic effects of ezrin require a phosphorylation-induced transition from oligomers to monomers at the plasma membrane. J. Cell Biol..

[227] Khanna C., Wan X., Bose S., Cassaday R., Olomu O., Mendoza A., Yeung C., Gorlick R., Hewitt S.M., Helman L.J. (2004). The membrane-cytoskeleton linker ezrin is necessary for osteosarcoma metastasis. Nat. Med..

[228] Chuan Y.C., Pang S.T., Cedazo-Minguez A., Norstedt G., Pousette A., Flores-Morales A. (2006). Androgen induction of prostate cancer cell invasion is mediated by ezrin. J. Biol. Chem..

[229] Song Y., Ma X., Zhang M., Wang M., Wang G., Ye Y., Xia W. (2020). Ezrin Mediates Invasion and Metastasis in Tumorigenesis: A Review. Front. Cell Dev. Biol..

[230] Bulut G., Hong S.H., Chen K., Beauchamp E.M., Rahim S., Kosturko G.W., Glasgow E., Dakshanamurthy S., Lee H.S., Daar I. (2012). Small molecule inhibitors of ezrin inhibit the invasive phenotype of osteosarcoma cells. Oncogene.

[231] Çelik H., Bulut G., Han J., Graham G.T., Minas T.Z., Conn E.J., Hong S.H., Pauly G.T., Hayran M., Li X. (2016). Ezrin Inhibition Up-regulates Stress Response Gene Expression. J. Biol. Chem..

[232] Çelik H., Hong S.H., Colón-López D.D., Han J., Kont Y.S., Minas T.Z., Swift M., Paige M., Glasgow E., Toretsky J.A. (2015). Identification of Novel Ezrin Inhibitors Targeting Metastatic Osteosarcoma by Screening Open Access Malaria Box. Mol. Cancer Ther..

[233] Ghaffari A., Hoskin V., Turashvili G., Varma S., Mewburn J., Mullins G., Greer P.A., Kiefer F., Day A.G., Madarnas Y. (2019). Intravital imaging reveals systemic ezrin inhibition impedes cancer cell migration and lymph node metastasis in breast cancer. Breast Cancer Res..

[234] Xue Y., Bhushan B., Mars W.M., Bowen W., Tao J., Orr A., Stoops J., Yu Y., Luo J., Duncan A.W. (2020). Phosphorylated Ezrin (Thr567) Regulates Hippo Pathway and Yes-Associated Protein (Yap) in Liver. Am. J. Pathol..

[235] Moodley S., Lian E.Y., Crupi M.J.F., Hyndman B.D., Mulligan L.M. (2020). RET isoform-specific interaction with scaffold protein Ezrin promotes cell migration and chemotaxis in lung adenocarcinoma. Lung Cancer.

[236] Hoskin V., Ghaffari A., Elliott B.E. (2019). Ezrin, more than a metastatic detERMinant?. Oncotarget.

[237] Hoskin V., Ghaffari A., Laight B.J., SenGupta S., Madarnas Y., Nicol C.J.B., Elliott B.E., Varma S., Greer P.A. (2022). Targeting the Ezrin Adaptor Protein Sensitizes Metastatic Breast Cancer Cells to Chemotherapy and Reduces Neoadjuvant Therapy-induced Metastasis. Cancer Res. Commun..

[238] Yu Y., Zeng P., Xiong J., Liu Z., Berger S.L., Merlino G. (2010). Epigenetic drugs can stimulate metastasis through enhanced expression of the pro-metastatic Ezrin gene. PLoS ONE.

